# Metabolic Crosstalk Between Host and Tumor as a Circuit of Resilience in Cancer Therapy

**DOI:** 10.3390/cells15111008

**Published:** 2026-05-30

**Authors:** Jingwen Wang, Hongyi Wu, Tianqi Wang, Meng Nie

**Affiliations:** 1 School of Public Health, Capital Medical University, Beijing 100069, China; jingwenwang0224@163.com; 2 School of Traditional Chinese Medicine, Capital Medical University, Beijing 100069, China; why0561ccmu@163.com; 3 Beijing Key Laboratory of Environment and Aging, Capital Medical University, Beijing 100069, China

**Keywords:** host, metabolism, cancer therapy

## Abstract

Therapeutic resistance in cancer arises not only from intrinsic metabolic plasticity within the tumor, but also from the systemic metabolic state of the host organism. This review advances an integrated framework centered on the metabolic network between the host and tumor to examine how host-related factors—particularly aging, nutrition, and psychological stress—remodel systemic metabolism and thereby influence the efficacy of diverse cancer therapies. We highlight a bidirectional metabolic interplay: host physiology establishes a permissive context for tumor metabolic adaptation, whereas anticancer therapies, in turn, perturb host metabolic homeostasis, accelerating aging and compromising neurocognitive health. Ultimately, we propose that overcoming therapeutic resistance will require strategies that simultaneously target tumor metabolic dependencies and reprogram the host metabolic milieu—a systemic approach poised to redefine precision oncology.

## 1. Introduction

Therapeutic resistance remains the principal obstacle to curative cancer therapy. Although decades of research have highlighted tumor-intrinsic mechanisms—including genetic mutations, epigenetic alterations and metabolic plasticity—that enable cancer cells to evade treatment, such cell-autonomous processes cannot fully account for the pronounced heterogeneity in patient responses. This discrepancy points to a largely underappreciated determinant of therapeutic outcome: the systemic metabolic state of the host.

Since the Warburg effect established the characteristic glucose metabolic signature of tumors, studies of tumor metabolic adaptation have expanded considerably [1,2]. Tumor cells rely on a highly flexible metabolic network to sustain survival and proliferation, enabling them to resist diverse therapeutic pressures [3,4]. Beyond cancer cell-intrinsic reprogramming, metabolic plasticity is shaped across interconnected compartments: the tumor itself, the tumor microenvironment (TME), and host systemic metabolism. Within the TME, stromal and immune cells engage in extensive metabolic crosstalk, modulating immune surveillance and treatment sensitivity, while lysosomal dysfunction and other TME-intrinsic circuits have been proposed to amplify resistance [5,6,7], albeit with inconsistent results likely attributable to host variables such as age and body composition.

Cancer metabolism does not act in isolation, but is embedded within and reciprocally regulated by host systemic metabolism [8]. Host factors including aging, nutritional status and psychological stress remodel circulating metabolites, hormonal axes and immune function, creating a metabolic milieu that either supports or restricts tumor progression under therapy [9]. Other host factors, such as physical activity [10], circadian rhythms [11], and obesity [12], similarly influence this systemic metabolic environment, although in this Review we focus primarily on aging, diet, and psychological stress. Strikingly, this crosstalk is bidirectional: anticancer treatments themselves disrupt host metabolic homeostasis, accelerating aging and impairing systemic function.

Most current investigations dissect tumor metabolism and host physiology separately, leading to a fragmented understanding of resistance. In this Review, we present an integrated framework of host–tumor metabolic interplay to address this gap. We outline how systemic host metabolism intersects with local tumor adaptation to drive resistance, discuss how aging, nutrition, and stress modulate this axis, and survey emerging therapeutic strategies targeting metabolic pathways, lifestyle interventions, and neuroendocrine signaling. We highlight that overcoming therapeutic resistance demands a shift from tumor-centric to systemic approaches, integrating both tumor dependencies and host metabolic status to advance precision oncology.

## 2. Metabolic Mediation of Host Factors in Therapy Response

**Figure 1:**
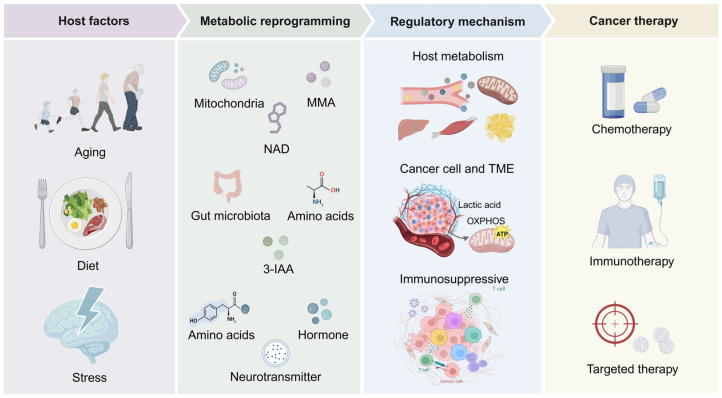
Host factors remodel systemic metabolism to influence therapy response.

Host factors—notably aging, nutritional status, and psychological stress—profoundly remodel systemic metabolism and dictate the efficacy of cancer therapy. In this section, we dissect how each of these factors reshapes the host metabolic landscape to promote therapeutic resistance (Figure 1).

### 2.1. Aging

Aging establishes the fundamental metabolic baseline of the host. The median age at diagnosis for most malignant tumors is over 60 years, and this proportion is expected to continue increasing over the next several decades [13,14,15]. With advancing age, the progressive accumulation of senescent cells and widespread systemic metabolic remodeling directly alters responses to diverse anticancer therapies. Yet traditional oncology research has largely neglected the systemic impact of aging biology on treatment efficacy and resistance [16].

Aging-associated metabolic reprogramming forms a critical driver of chemotherapy resistance. Gomes et al. demonstrated that serum methylmalonic acid (MMA) accumulates substantially with age, inducing expression of the transcription factor SOX4 to trigger epithelial–mesenchymal transition and stemness in tumor cells. This not only enhances metastatic potential but also confers resistance to conventional chemotherapies including carboplatin and paclitaxel [17]. Beyond circulating metabolites, aging amplifies therapy-induced senescence, further reinforcing resistance. Mechanistically, platinum-induced senescence elicits a senescence-associated secretory phenotype (SASP) enriched in TGFβ, which activates the TGFBR1–AKT/mTOR axis and enhances mitochondrial respiration. This rewiring provides tumor cells with a metabolic advantage for energy production and stress tolerance, culminating in cisplatin resistance. Notably, in middle-aged mice recapitulating human aging, the senescent lung microenvironment markedly potentiates SASP-driven metabolic remodeling, leading to far stronger drug resistance than in young animals [18]. These findings reinforce that age strongly modulates metabolite–tumor crosstalk.

In targeted therapy, the aged host microenvironment reshapes stromal cell metabolism to blunt treatment responses. Aging remodels lipid metabolism in dermal fibroblasts, driving secretion of ceramide-enriched lipids that are taken up by melanoma cells via Fatty Acid Transport Protein 2 (FATP2) to sustain mitochondrial metabolism. This supports energy supply and stress resistance, reducing sensitivity to BRAF/MEK inhibition in elderly patients [19,20]. Importantly, aging-associated metabolic rewiring exhibits pronounced sex heterogeneity. Aged male hosts develop greater resistance to BRAF/MEK inhibitors via reactive oxygen species (ROS)-driven BMP2 secretion, whereas aged females display distinct metabolic vulnerabilities linked to declining estrogen levels—dimensions rarely addressed in existing reviews, limiting translational precision. Recent work further revealed that the aged systemic milieu drives a male-specific ROS imbalance that promotes BMP2 secretion, triggering metabolic reprogramming in melanoma cells that enhances invasiveness and drug resistance. This uncovers a unique metabolic vulnerability underlying inferior clinical outcomes in elderly male patients [21].

Aging-related metabolic dysregulation also severely impairs immunotherapy efficacy. On one hand, aging disrupts Nicotinamide adenine dinucleotide (NAD^+^) metabolism and mitochondrial function, contributing to age-related declines in Chimeric Antigen Receptor T-cell (CAR-T) therapeutic activity [22]. On the other hand, age-associated reductions in adipocyte-derived leptin accelerate senescence of tumor-infiltrating CD8^+^ T cells through the p38 pathway, impairing antitumor immunity and blunting immunotherapy responses [23]. Aging-induced immunometabolic disturbances are key determinants of immune dysfunction and the ‘cold tumor’ microenvironment. Deeper mechanistic dissection of how aging shapes therapy responses via immunometabolic regulation may yield new insights into age-dependent disparities in immunotherapy efficacy and enable strategies to overcome resistance [24,25].

### 2.2. Diet

Diet, the most dynamically modifiable host factor, has emerged as a focal point of breakthrough research in cancer therapy resistance. Unlike irreversible genetic backgrounds, diet shapes therapeutic response by altering circulating metabolite pools, remodeling the TME metabolic landscape, and modulating drug pharmacokinetics—offering actionable avenues to mitigate resistance. In this section, we examine three levels through which diet affects cancer therapy: dietary patterns shape nutrient composition and systemic metabolism; caloric or nutrient restriction limits energy or nutrient intake to induce metabolic stress and immune/tumor cell reprogramming; and diet-derived metabolites act as molecular mediators of therapy sensitivity.

Among specific dietary patterns, the ketogenic diet (KD) and the Mediterranean diet (MedDiet) are particularly notable as clinically feasible holistic dietary interventions. To illustrate the potential of these approaches, we first focus on KD, which has been extensively studied for its effects on tumor metabolism and therapeutic response. KD reshapes tumor metabolism and immune microenvironment via fuel supply changes. Preclinically, it exerts antitumor effects via microbiota modulation, anti-tumor microglia [26], or BHB-mediated CAR-T persistence [27], with tumor-specific variability. In a mouse model of pancreatic ductal adenocarcinoma, KD alone had minimal impact on tumor growth, but when combined with cytotoxic chemotherapy (nab-paclitaxel, gemcitabine, cisplatin), it synergistically inhibited tumor growth and tripled survival benefits [28]. Unexpectedly, KD synergizes with PI3K inhibitors not via macronutrient composition, but by reducing dietary phytochemicals (e.g., soy saponins); this relieves gut microbiota-induced hepatic drug-metabolizing enzyme expression, increasing drug exposure and therapeutic efficacy [29]—highlighting the need to consider microbial metabolism of dietary components in therapeutic design. In contrast, the MedDiet, rich in fruits, vegetables, whole grains, olive oil, and fish, can modulate systemic metabolism, reduce inflammation, and enhance immune function, thereby providing a supportive environment for conventional therapies.

Beyond specific dietary patterns, caloric and nutrient restriction strategies, including continuous dietary restriction (DR), short-term fasting, and fasting-mimicking diets (FMDs), modulate antitumor immunity and therapeutic responsiveness by inducing systemic nutrient stress and metabolic reprogramming. Conceptually, continuous DR involves sustained caloric reduction without malnutrition, whereas short-term fasting and FMDs impose transient nutrient deprivation, with FMDs designed to reproduce the metabolic effects of fasting while improving clinical tolerability. DR slows tumor growth by optimizing CD8^+^ T cell function within the TME, in part via increased circulating β-hydroxybutyrate, which reprograms T cell metabolism and fate, and synergizes with anti-PD-1 immunotherapy to enhance antitumor responses [30]. Short-term starvation similarly augments the efficacy of PD-1 blockade in mice, primarily by reducing circulating insulin-like growth factor 1 (IGF-1) and downregulating IGF-1 receptor signaling in tumor cells; clinically, elevated plasma IGF-1 or high tumor IGF-1R expression correlates with resistance to anti-PD-1/PD-L1 therapy [31]. Periodic fasting promotes antitumor immunity through natural killer (NK) cell-dependent metabolic reprogramming: NK cells migrate to the bone marrow, upregulate Carnitine Palmitoyltransferase 1A (CPT1A) to enhance fatty acid oxidation and survival, and are further activated by glucocorticoids in cooperation with myeloid cell-derived IL-12, leading to increased IFN-γ production [32]. Collectively, these interventions converge on the regulation of systemic energy sensing, yet utilize distinct immune-metabolic pathways to enhance antitumor activity. Preclinical studies also indicate that FMDs can synergize with conventional therapies, including radiotherapy, chemotherapy, and immunotherapy [31,33], highlighting their translational potential in combination treatment strategies.

In addition to overall dietary patterns and caloric/nutrient interventions, individual dietary components—specifically amino acids and their metabolites, as well as dietary bioactive molecules—serve as direct micro-level mediators of therapy sensitivity, forming the molecular basis of dietary effects. Tryptophan, an essential dietary amino acid, is a paradigmatic example: gut microbiota-derived tryptophan metabolite indole-3-acetic acid (3-IAA) enhances pancreatic cancer chemotherapy efficacy, with higher serum 3-IAA correlating with better patient responses; fecal microbiota transplantation, short-term dietary tryptophan modulation, or oral 3-IAA administration enhanced FOLFIRINOX efficacy in humanized germ-free pancreatic cancer mice, dependent on neutrophil-derived myeloperoxidase [34]. Similarly, the probiotic *Lactobacillus reuteri* colonizes melanoma tumors, releasing dietary tryptophan metabolite indole-3-aldehyde to boost IFN-γ-producing CD8^+^ T cells and enhance immune checkpoint inhibitor efficacy [35], while dietary tryptophan-derived AhR ligands (e.g., indole-3-carbinol [I3C], diindolylmethane [DIM]) are prerequisites for optimal PD-1 inhibitor efficacy [36]. Beyond tryptophan, histidine metabolism is closely linked to methotrexate resistance [37], further expanding the micro-level molecular mechanisms of dietary modulation.

Recent metabolomics research in esophageal squamous cell carcinoma further confirms the role of micro-level dietary bioactive molecules: dietary-derived bioactive compounds—S-allyl-L-cysteine from garlic and indole-3-carbinol from cruciferous vegetables—correlated with better chemoimmunotherapy responses by promoting NK cell infiltration and reversing CD8^+^ T cell exhaustion [38]. Dietary patterns also shape resistance via additional micro-level pathways, including modulation of oral targeted agent bioavailability, dietary fiber intake, and fructose metabolism [39,40,41,42,43]. Collectively, diet acts as a flexible, accessible tool to complement cancer therapies, operating via a hierarchical mechanism—from macro dietary patterns to micro molecular metabolites—with the diet-microbiota-immunity axis as a central regulatory hub. Preclinical-clinical translational gaps (e.g., strict dietary control in models vs. patient adherence challenges, limited clinical data) and individual variability (sex, age, baseline microbiota) remain key barriers to personalized dietary strategies—critical areas for future research to integrate dietary modulation into precision oncology.

### 2.3. Stress

Psychological stress represents a prevalent and modifiable host factor with profound impacts on cancer development, progression and patient prognosis. Accumulating evidence has linked chronic stress—including social, physical, and emotional stressors [44]—to accelerated tumor progression, therapy resistance and poor clinical outcomes, as highlighted in major reviews across cancer biology, immunology, and metabolism [45,46,47,48]. Importantly, emerging mechanistic work has revealed that stress does not merely promote tumor growth, but establishes a therapy-refractive niche by coordinately remodeling systemic host metabolism and local tumor metabolic networks. A central unresolved question is how neuroendocrine stress signals are transduced into metabolic programmings that directly govern therapeutic response.

At a systemic level, psychological stressors such as social defeat and emotional distress trigger marked elevations in glucocorticoids, which drive dual metabolic and immune inhibitory programs. Glucocorticoid signaling acts directly through the stress–glucocorticoid–TSC22D3 axis to suppress antitumor immunity, while chronic excess leads to systemic metabolic dysfunction including hyperglycemia and muscle catabolism, further exacerbating metabolic stress in immune cells and undermining therapeutic efficacy [49]. Glucocorticoid receptor activation also enables disseminated tumor cells to evade CD8^+^ T cell and NK cell killing by repressing FAS expression [50]. In clinical cohorts, emotional distress—including depression and anxiety—correlates with elevated cortisol, reduced response rates and shorter progression-free survival in patients with advanced non-small cell lung cancer receiving immune checkpoint inhibitors [51]. Beyond glucocorticoids, catecholamines from the sympathetic nervous system propagate stress signaling: in radiotherapy models, β2-adrenergic signaling suppresses antitumor immunity, diminishing local efficacy and the abscopal effect [52]. Additionally, stress-induced pregnenolone elevation promotes tumor progression and immunotherapy resistance, revealing an underappreciated link between behavioral stress, steroid metabolism, and immune evasion [53].

In parallel to systemic changes, stress hormones act directly on tumor cells to drive cell-autonomous metabolic reprogramming, which synergizes with systemic metabolic disturbance to form a therapy-resistant microenvironment. In colorectal cancer, chronic stress activates the β2-AR/PKA/CREB1 axis, upregulating key glycolytic enzymes including GLUT1, Hexokinase 2 (HK2) and Phosphofructokinase, Platelet type (PFKP) to enhance glycolytic flux and support proliferation and survival [54]. In liver cancer, glucocorticoid-induced NR3C1 nuclear translocation transcriptionally activates Alanyl Aminopeptidase (*ANPEP*), remodeling amino acid metabolism and boosting glutathione synthesis to inhibit ferroptosis and confer sorafenib resistance [55]. Stress also mediates tissue-specific immunometabolic remodeling: in the liver, β2-AR signaling represses Quinolinate Phosphoribosyltransferase (QPRT) in hepatocytes, shunting kynurenine metabolism toward kynurenic acid accumulation, impairing mitochondrial function and CD8^+^ T cell effector activity [56]. These local metabolic alterations propagate across the TME via extracellular vesicles and signaling molecules. In ovarian cancer, the stress-associated neurotransmitter serotonin polarizes tumor-associated macrophages to secrete extracellular vesicles (EVs) enriched in PI4K2A and ITPKC. These inositol-metabolic enzymes elevate nuclear inositol-1,3,4,5-tetrakisphosphate, activating MRE11 to enhance homologous recombination repair and drive resistance to cisplatin and PARP inhibitors [57]. Stress signals also remodel tumor innervation and lymph node immune status, further disrupting neuro-immune-tumor crosstalk [58,59].

As critical executors of therapeutic response, immune cells are profoundly dysregulated by stress-induced metabolic reprogramming. Chronic stress directly impairs CD8^+^ T cell effector function and increases PD-1 expression through β2-AR signaling, blunting the efficacy of anti-PD-1 immune checkpoint blockade [60]. Beyond host and tumor cells, stress integrates gut microbiota metabolism into this regulatory network, adding another layer of context dependence that complicates translational efforts [61].

Notably, neural-cancer crosstalk has emerged as a rapidly expanding frontier in stress-mediated tumor progression [62,63,64,65,66]. Bidirectional communication between the nervous system and tumors likely serves as a key conduit through which psychological stress shapes therapeutic resistance, particularly via sympathetic nervous system activation. However, this integrated neuro-endocrine-metabolic-immune axis remains poorly incorporated into current resistance models, representing a major conceptual and translational gap in the development of host-centered cancer therapies.

## 3. Metabolic Remodeling of Host Physiology by Therapies

**Figure 2:**
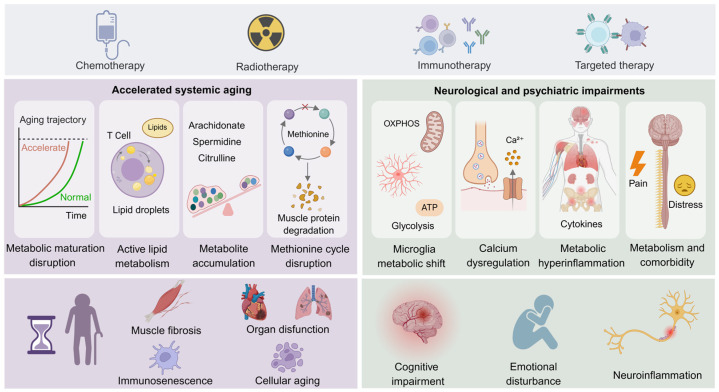
Therapy-induced metabolic reprogramming drives accelerated aging and neurocognitive impairment in host tissues.

Anticancer therapies, while targeting tumor cell elimination, inadvertently induce profound metabolic reprogramming in normal host tissues, triggering long-term physiological perturbations that extend beyond direct treatment toxicity. This review dissects two interconnected manifestations of therapy-induced metabolic dysregulation—accelerated host aging and neurocognitive impairment—highlighting their shared metabolic underpinnings, tissue-specific mechanisms, and unmet translational gaps (Figure 2). Critically, these host metabolic alterations not only compromise long-term survivor quality of life but also create a permissive microenvironment for tumor recurrence and therapy resistance, forming a bidirectional regulatory loop between treatment efficacy and host physiology.

### 3.1. Cancer Therapies Accelerate Host Aging

The aged metabolic microenvironment constrains therapeutic efficacy, but an underappreciated reciprocal relationship exists: cancer therapies themselves act as potent catalysts of host aging through targeted disruption of core metabolic pathways in normal tissues. As the cancer survivor population expands, therapy-induced aging and its associated comorbidities have emerged as critical determinants of long-term outcomes, yet the metabolic mechanisms linking treatment to accelerated aging remain incompletely integrated into resistance models.

Aging is defined by cumulative multidimensional biological damage, with hallmarks interconnected via complex molecular networks. Chemotherapy, radiotherapy, and immunotherapy—while clinically essential—non-specifically perturb systemic metabolic homeostasis, triggering a cascade of aging-related pathologies: somatic mutation accumulation, cellular senescence, immune exhaustion, and organ-specific dysfunction. Notably, TIA exhibits striking tissue specificity, driven by inherent metabolic differences across organs. Deep sequencing of normal tissues from multiple organs reveals that treatment-induced somatic mutations are enriched in tissues with high metabolic demands, providing a molecular basis for the heterogeneous aging rates observed across organ systems [67].

This tissue specificity is further modulated by life stage: in childhood and adolescent survivors, chemotherapy-induced metabolic damage occurs during critical growth and developmental windows, disrupting normal metabolic maturation. This not only upregulates the senescence marker p16INK4a but also accelerates telomere shortening and epigenetic aging via DNA damage, oxidative stress, and epigenetic dysregulation—trajectories that permanently alter physiological, biological, and cognitive aging [68,69,70].

Treatment-induced senescence is not cell-autonomous but propagates systemically via metabolic crosstalk, enabling cross-organ damage dissemination. Radiotherapy, for example, triggers arachidonic acid accumulation in tumor cells and packages spermidine synthase into EVs; these EVs deliver spermidine to skeletal muscle, promoting eIF5A-dependent type I collagen biosynthesis and ultimately muscle fibrosis and weakness [71]. In the central nervous system (CNS), radiation-induced senescence in astrocytes drives secretion of SASP factors, activating the Met metabolic pathway in glioma cells and exacerbating brain aging and functional decline [72]. Similarly, methionine cycle disruption during cancer cachexia induces skeletal muscle DNA hypomethylation and endoplasmic reticulum stress, upregulating REDD1 and activating autophagy/ubiquitin-proteasome pathways to accelerate muscle protein degradation [73]—a manifestation of local metabolic imbalance amplifying systemic aging.

The immune system serves as a key mediator of TIA, with treatment-induced metabolic alterations driving immune senescence that propagates systemic aging. Tumor-derived EVs with high PD-L1 expression induce T cell DNA damage and robust lipid metabolism dysregulation, increasing cholesterol accumulation and lipid droplet formation; this activates CREB/STAT signaling to promote T cell senescence [74]. Therapy-resistant tumor cells further exploit metabolic signals to impair CD8^+^ T cell energy metabolism, weakening antitumor immunity while accelerating immune senescence—changes strongly correlated with relapse and poor prognosis [75]. In rectal cancer, radiotherapy activates the IL-1α metabolic pathway, polarizing cancer-associated fibroblasts toward an inflammatory phenotype and inducing p53-mediated senescence, linking chemoradiotherapy resistance to tissue aging [76]. Critically, selective induction of immune cell senescence (via Ercc1 deletion in hematopoietic cells) accelerates aging in non-lymphoid organs, while transplanting young immune cells alleviates systemic aging—demonstrating immune metabolism as a central driver of TIA [77].

Tumors synergize with therapy-induced metabolic damage to amplify aging, forming a vicious cycle: tumor-derived metabolic signals exacerbate treatment-induced host aging, which in turn reinforces tumor progression and resistance [78]. For example, lung cancer-derived Dimethylarginine Dimethylaminohydrolase 1 (DDAH1) induces abnormal citrulline accumulation, inhibiting TGF-β1 citrullination and activating the TGF-β1/Smad3 pathway to accelerate pulmonary fibrosis and lung aging. When combined with radiotherapy/chemotherapy-induced lung damage, this significantly worsens pulmonary function and long-term complication risk [79]. Persistent therapy-induced senescent cells further mediate chronic inflammation via metabolic dysregulation, contributing to side effects such as bone marrow suppression, cardiac dysfunction, and physical decline [80]—underscoring the need to target the metabolism–aging network to improve survivor outcomes.

### 3.2. Therapies Influence Cognition

Therapy-induced metabolic reprogramming extends beyond aging to disrupt CNS function, driving neuropsychiatric impairments, such as cognitive dysfunction and emotional disturbances, that limit survivor quality of life. Neurotoxicity, a common adverse effect of anticancer therapies, disrupts central and peripheral nervous system function [81], while cancer-related psychological distress—exacerbated by treatment-induced metabolic changes—increases suicide risk and long-term mortality [82,83]. Clinical cohorts confirm that therapies induce persistent, pattern-specific cognitive impairment, closely linked to emotional status and treatment-related metabolic disruptions [84,85,86].

Chemotherapy-induced cognitive impairment (“chemobrain”)—long mechanistically elusive—has clear metabolic origins, yet these are rarely integrated into resistance models. Classic chemotherapeutics (5-fluorouracil, methotrexate, doxorubicin) independently induce cognitive deficits across cancer types [87,88,89]. For example, doxorubicin activates protein kinase A to induce abnormal phosphorylation, oxidation, and nitrosylation of neuronal ryanodine receptor type 2 (RyR2), depleting its stabilizer calstabin2 and causing calcium leakage. This disruption of neuronal calcium homeostasis directly impairs brain glucose metabolism, driving neurocognitive dysfunction [90]—linking systemic metabolic perturbation to CNS-specific damage.

Immunotherapies have further complicated the landscape of treatment-related neurotoxicity. CAR-T cell therapy, despite its efficacy, carries significant neurotoxic risk: it induces persistent CNS microglial reactivity, with activated microglia shifting from oxidative phosphorylation to glycolysis, mediating neuroinflammation and cognitive impairment [91]. Immune effector cell-associated neurotoxicity syndrome (ICANS), a severe CNS complication of immune-activating therapies, presents with core cognitive deficits correlated with blood–brain barrier disruption and immune cell infiltration. In treatment-refractory severe ICANS, cerebrospinal fluid exhibits CAR-T cell enrichment, non-CAR-T clone hyperproliferation [92], and a persistent immunometabolic storm—suggesting refractory T cell clones drive uncontrolled CNS metabolic inflammation. This storm is amplified by systemic metabolic crosstalk: T cell-activating therapies induce cytokine release and a surge in mood-associated catecholamines, which trigger a self-amplifying loop in macrophages to exacerbate immune dysregulation and cytokine storm [93].

Tumor–neuroendocrine interactions further contribute to cognitive and emotional disorders via shared metabolic pathways that link neuropsychiatric symptoms to therapy resistance—a critical unmet research gap. In a pancreatic ductal adenocarcinoma mouse model, tumor-derived S-adenosylmethionine (SAM) upregulates methyltransferase-like protein 14 in glutamatergic neurons, enhancing neuronal excitability via m6A modification and mediating pain–depression comorbidity. A methionine-restricted diet reduces circulating SAM, alleviating neurobehavioral deficits and inhibiting tumor growth [94]—revealing that neuropsychiatric symptoms and resistance are driven by overlapping metabolic pathways. Current models fail to integrate these connections, limiting our ability to target both outcomes simultaneously.

Collectively, anticancer therapies induce systemic metabolic reprogramming that drives two interconnected host phenotypes—accelerated aging and neurocognitive impairment—via shared mechanisms (metabolic dysregulation, senescence, immune dysfunction) and tissue-specific pathways. The bidirectional loop between therapy-induced metabolic damage, host aging, neurocognitive decline, and tumor resistance remains understudied, with critical gaps in understanding how to target metabolic nodes to improve both treatment efficacy and survivor quality of life. Future research must move beyond isolated mechanistic studies to integrate these phenotypes into a unified metabolic framework, addressing translational challenges such as tissue-specific vulnerability and personalized metabolic intervention.

## 4. Therapeutic Interventions in Host Metabolism

**Figure 3:**
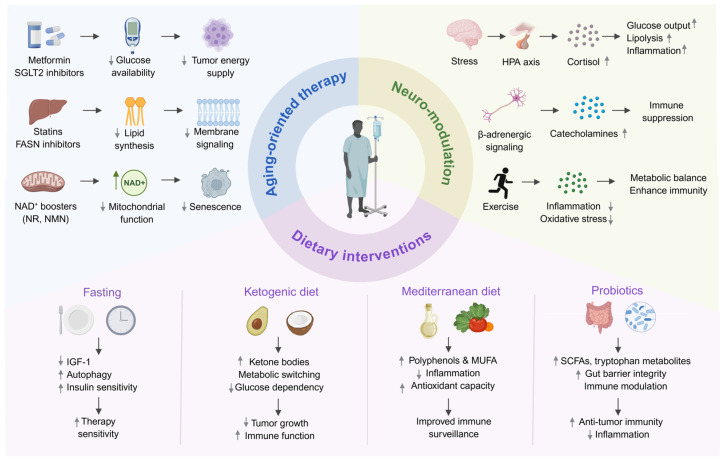
Host metabolism as a targetable hub for improving therapeutic efficacy.

Based on our unified framework of host-tumor metabolic crosstalk, overcoming therapy resistance requires synergistic strategies targeting both tumor metabolic dependencies and the host metabolic milieu (Figure 3). Host metabolism—shaped by aging, nutrients, neuroendocrine signaling, and microbiota—is a modifiable intervention platform, with targeted and behavioral therapies holding translational potential to optimize efficacy and long-term survivor outcomes. Below, we analyze key interventions by metabolic pathway and regulatory axis, focusing on their clinical translational applications and discussing existing clinical evidence and practical challenges for optimizing therapy.

### 4.1. Aging: The Metabolic Context for Intervention

Aging-driven metabolic dysregulation creates a therapy-resistant microenvironment, making anti-aging metabolic interventions foundational for elderly cancer patients. Targeting core pathways (sugar, lipid, amino acid metabolism) and key molecules (vitamins, NAD^+^) reverses age-related systemic dysfunction and sensitizes tumors to therapy, addressing the bidirectional aging–treatment response link.

#### 4.1.1. Glucose Metabolism

Glucose metabolism links host aging and cancer therapy response. Metformin, a widely used glucose-lowering agent, delays aging in animals by improving cognitive function in aged mice [95] and reversing primate brain aging by ~6 years, via gut microbiota modulation, inflammatory suppression, and blocking abnormal chromatin transport in senescent cells [96]. In type 2 diabetes patients, 26 weeks of henagliflozin, a novel SGLT2 inhibitor, extended telomere length, enhanced T cell function, and remodeled metabolism—suggesting multi-pathway anti-aging effects [97].

In cancer, metformin weakens *DNMT3A-R882* mutant hematopoietic stem cell clonal advantage reducing Acute Myeloid Leukemia transformation risk [98,99] and enhances γδ T cell tumor-killing, amplified by lower blood glucose [100]. A target trial emulation showed SGLT2 inhibitors reduced treatment failure and delayed progression in prostate cancer patients on androgen deprivation therapy, beyond glucose control. Conversely, metformin did not benefit non-diabetic abdominal aortic aneurysm patients, though sample size was limited [101]. Sugar metabolism interventions have dual anti-aging/anticancer potential for elderly patients, but require stratification by baseline glucose metabolism and comorbidities.

#### 4.1.2. Lipid Metabolism

Aging-related systemic lipid dysregulation exacerbates therapy resistance via immune suppression, tumor stemness, mutant p53 stabilization, and metabolic feedback loops [102,103,104,105,106]. Affecting multiple tissues, including heart [107], blood vessels [103], and cartilage [108], it is driven by 3-Hydroxy-3-Methylglutaryl-CoA Reductase (HMGCR), SREBP1, and fatty acid synthase (FASN)—actionable targets.

Statins (HMGCR inhibitors) reverse chemotherapy resistance: in advanced pancreatic cancer, combinations achieved 70.3% clinical benefit [109]. SREBP1 drives metabolic feedback loops, correlating with poor pancreatic cancer prognosis and mediating immune evasion via SREBP1-PCSK9-PD-L1 [110]. FASN inhibition impairs DNA repair and enhances chemotherapy sensitivity, supported by a colorectal cancer window-of-opportunity trial [111]. Although these trials were not specifically designed for elderly patients, the potential benefits of such interventions for older cancer patients with age-related lipid metabolism disorders are worth investigating.

#### 4.1.3. Amino Acid Metabolism

Methionine metabolism connects aging and cancer therapy. Common in aging, methionine restriction extends animal lifespan and reverses age-related dysfunction [112,113]. Although an 8-week human clinical trial showed that methionine restriction did not have a significant effect on the epigenetic clock, the study still provides preliminary support for the translational application of methionine restriction in humans [114]. In the context of cancer, methionine metabolic interventions exhibit seemingly paradoxical and multifaceted effects, and their underlying mechanisms remain under active investigation. The outcomes of these interventions are highly dependent on the mode and timing of intervention, as well as the complex interactions among different cell types [115,116,117]. Methionine restriction was well tolerated in a dietary intervention study involving healthy volunteers [117], but strict dietary regimens lead to poor patient adherence and remain a major obstacle to broader application [118].

A major limitation is the one-size-fits-all approach: methionine restriction enhances response in some tumors but promotes resistance in others, depending on tumor type and host nutrition. Future strategies require stratification by host metabolic signatures (e.g., NAD^+^ levels, lipid profiles, microbiota). Glutamine metabolism is another target: aging-related enhanced catabolism drives senescence [119,120]. Telaglenastat, a glutaminase inhibitor, plus azacitidine achieved 70% response in advanced Myelodysplastic Syndromes [121] but failed in metastatic Renal Cell Carcinoma [122]. Notably, the aged microenvironment induces glutamine dependence in aged lung cancer via ISR-ATF4, driving metastasis; targeting this suppresses invasion [123]—supporting use in elderly patients.

Similarly, taurine, which declines with aging, has shown potential in reversing age-related dysfunction, yet its role in both aging and cancer remains controversial, highlighting the need for further mechanistic studies and clinical trials [124,125,126,127,128,129].

#### 4.1.4. Vitamins and Micronutrients

Vitamins/micronutrients have evolved from nutritional support to precision therapies. A large randomized trial found daily multivitamins slowed epigenetic aging, especially in those with baseline acceleration [130]. In cancer, high-dose IV vitamin C plus gemcitabine/nab-paclitaxel extended metastatic pancreatic cancer OS from 8.3 to 16 months [131], targeting both aged host metabolism and tumor vulnerabilities.

Vitamin D supplementation reduces overall cancer mortality [132], with higher levels correlating with improved anti-PD-1 response and reduced immune-related colitis in melanoma [133,134]. Niacin (B3) and Nicotinamide (NAM) show promise: controlled-release niacin improved glioblastoma 6-month PFS by 28% [135], while NAM-expanded NK cells (GDA 201) achieved 74% ORR in relapsed/refractory NHL [136]. Individualization by host status, tumor type, and regimen is critical.

#### 4.1.5. NAD^+^ and Spermidine

Age-related systemic NAD^+^ decline drives aging-related diseases [137] via organ-specific mechanisms: early ovarian depletion accelerates aging [138]. CNS REV-ERBα upregulation impairs NAD^+^ homeostasis and astrocyte function [139] and the liver–kidney NaR axis declines with age [140].

Restoring NAD^+^ reverses age-related metabolic decline, restores immune function, and enhances antitumor immunity [141,142]. Clinically, oral nicotinamide riboside (NR) improves vascular function in older adults [143]; daily nicotinamide mononucleotide increases whole-blood NAD^+^ and physical function in elderly men [144]; NR reduces sputum IL-8 and improves epigenetic aging in COPD [145]. NAM-expanded NK cells plus rituximab achieved 74% ORR in relapsed/refractory NHL, with sustained NK cell metabolic fitness [136]. NAD^+^ targeting serves as both anti-aging and anticancer adjuvant.

Other metabolites, such as spermidine, have garnered attention in both aging and cancer research [71,146,147,148,149,150,151] and may represent a promising candidate for modulating the interplay between host aging and antitumor therapy. In cancer contexts, its role remains context-dependent, necessitating careful evaluation of host metabolic status and tumor biology to harness its therapeutic potential without unintended support of tumor growth.

### 4.2. Dietary Interventions

Dietary interventions reprogram host metabolism as feasible anticancer adjuvants. Fasting, KD, MedDiet, and nutrient supplements are transitioning to clinical use, with efficacy varying by tumor type, regimen, and adherence.

#### 4.2.1. Fasting

Periodic fasting and FMDs have shown encouraging clinical feasibility and translational potential in cancer therapy. In a 101-patient clinical trial, FMD was well tolerated and was associated with reduced circulating glucose and growth factor levels together with systemic immune remodeling [152]. In early Triple-Negative Breast Cancer, the addition of FMD to preoperative chemotherapy improved pathological complete response (pCR) rates [153]. Moreover, preliminary clinical observations in colorectal cancer patients undergoing surgery suggested that 16 h short-term preoperative fasting could reshape the tumor immune microenvironment and promote a more cytotoxic, less exhausted CD8^+^ T-cell phenotype [154]. Long-term adherence remains a challenge, especially in elderly patients with comorbidities.

#### 4.2.2. Ketogenic Diet

In humans, a 2-week KD modulates immunity [155]. Clinical studies suggest that KD reduces proinflammatory factors and insulin levels, creating a favorable systemic metabolic environment while also improving patient function and promoting tumor regression [156,157]; in glioma, it is well tolerated, lowers glucose/insulin, and achieves brain ketones [158]. Strict macronutrient requirements limit utility, especially for elderly nutritional status.

#### 4.2.3. Mediterranean Diet

The moderate, sustainable MedDiet exerts antitumor effects via metabolic modulation and inflammation suppression. Population studies show inverse associations with cancer risk [159]. In gastric cancer, high adherence correlates with earlier stage, longer survival, and better quality of life [160]; in chemotherapy patients, it alleviates fatigue via improved T cell mitochondrial respiration [161], providing preliminary evidence-based support for the application of this dietary approach in tumor supportive care.

#### 4.2.4. Nutrients and Dietary Supplements

Specific nutrients/supplements enhance therapy via microbiota, immune, and metabolic modulation. Probiotics improve outcomes: Bifidobacterium CBM588 extended metastatic Renal Cell Carcinoma Progression-Free Survival [162] and TKI response [163]; Clostridium butyricum CBM588 improved Non-Small Cell Lung Cancer (NSCLC) survival, especially with antibiotics/PPIs [164,165]. Fecal microbiota transplantation shows potential for immunotherapy response/toxicity reduction, but larger trials are needed [166,167,168]. Together, these findings highlight how interactions between gut bacteria and the body’s metabolism may serve as an important target for supporting cancer treatment.

However, some dietary interventions still carry potential risks [169,170,171], and their long-term effects in patients with cancer remain incompletely understood. Therefore, although these strategies provide feasible approaches to enhance therapeutic efficacy, careful patient selection, monitoring, and clinical evaluation are still required. Future studies should systematically assess their safety, feasibility, and individualized implementation strategies.

### 4.3. Targeting Neural and Neuroendocrine Pathways

Neuroendocrine factors (e.g., psychological stress) induce resistance via metabolic and TME remodeling, making neural pathways a promising frontier. Targeting the neuroendocrine system (e.g., β-blockers, glucocorticoid antagonists) addresses stress-induced resistance, but limited mechanistic understanding and stratification criteria prevent standard care integration.

#### 4.3.1. Neurotransmitters and Related Pathways

Catecholamines released by the sympathetic nervous system are key molecules mediating stress-induced immunosuppression. The clinical translational value of this regulatory axis has been validated by several studies. A randomized, triple-blind, placebo-controlled phase II trial in patients with early-stage breast cancer showed that short-term preoperative use of the non-selective beta-blocker propranolol remodeled tumor transcriptomic profiles, downregulated pro-metastatic stromal gene expression, and promoted intratumoral CD8^+^ T cell infiltration [172]. However, population-based retrospective studies indicated that this benefit is molecular subtype-specific: beta-blocker use was associated with longer cancer-specific survival only in triple-negative breast cancer, with a hazard ratio of 0.66, a finding supported by meta-analysis [173]. In melanoma, a prospective study found that propranolol use after diagnosis in patients with stage IB-IIIA disease was associated with a significantly reduced risk of recurrence, approximately 80% [174]. Beyond these, existing clinical neuroactive drugs have also shown considerable repurposing potential. Selective serotonin reuptake inhibitors, a class of classic antidepressants, have been demonstrated in mechanistic studies to synergize with PD-1 blockade to exert antitumor effects [175]. Another clinically approved antidepressant, vortioxetine, exhibited potent antitumor activity in patient-derived glioblastoma specimens, offering a feasible pathway for rapid clinical translation of neuro–tumor interaction targets [176]. The association of cholinergic pathways with tumor progression and treatment resistance is also increasingly coming into focus. A retrospective analysis based on Norwegian national registry data showed that in patients with lung cancer, the use of antimuscarinic drugs after diagnosis was associated with longer lung cancer-specific survival, with a more pronounced association observed in patients receiving chemotherapy [177].

#### 4.3.2. Neuropeptides and Neurotrophins

Precision intervention in neural signaling pathways is emerging as a frontier in overcoming resistance to cancer therapy. Among these, strategies targeting neuropeptides and neurotrophin receptors have achieved breakthroughs in several tumor types. The first-generation tropomyosin receptor kinase inhibitor larotrectinib demonstrated rapid and durable efficacy in patients with primary central nervous system tumors harboring neurotrophic receptor tyrosine kinase gene fusions, with a median time to response of 1.9 months, a 24-week disease control rate of 73%, and a favorable safety profile [178]. However, acquired resistance represents a bottleneck limiting its long-term efficacy. The next-generation macrocyclic tropomyosin receptor kinase inhibitor repotrectinib was designed to overcome such resistance. Its registrational study enrolled patients with neurotrophic receptor tyrosine kinase gene fusion-positive solid tumors and showed a response rate of 59% in patients without prior tyrosine kinase inhibitor treatment, with a median progression-free survival of 30.3 months. In patients who had received prior tyrosine kinase inhibitor therapy, the response rate was 48%, and repotrectinib was effective in patients harboring solvent front mutations while also demonstrating considerable intracranial efficacy [179].

The neurokinin-1 receptor antagonist aprepitant, a classic antiemetic drug, has demonstrated clinical value beyond symptom management. A phase III randomized controlled trial showed that in young female patients with gastrointestinal cancers receiving FOLFIRI or FOLFOX chemotherapy, adding aprepitant to palonosetron and dexamethasone significantly improved the complete response rate from 66.7% to 87.0% [180]. More importantly, a retrospective analysis based on Norwegian national registry data revealed that aprepitant use during chemotherapy was significantly associated with improved distant disease-free survival and breast cancer-specific survival in patients with early-stage breast cancer, with the effect being more pronounced in non-luminal subtypes, particularly triple-negative breast cancer [181]. These findings suggest that drugs targeting neuropeptide receptors may possess antitumor value independent of their antiemetic effects.

#### 4.3.3. Hormonal Interventions

A phase III clinical trial in patients with platinum-resistant ovarian cancer provided key evidence for targeted intervention in hormone signaling pathways. The results showed that adding the selective glucocorticoid receptor antagonist relacorilant to standard chemotherapy (nab-paclitaxel) significantly extended median progression-free survival from 5.5 months to 6.5 months, and an interim analysis of overall survival also showed benefit (15.97 months vs. 11.50 months) [182]. This study provided the first large-scale clinical trial evidence that blocking downstream signaling pathways of stress-related hormones can effectively sensitize tumors to chemotherapy. It should be noted that glucocorticoids have a very complex role in cancer treatment. In some cases, they may enhance cancer cells’ ability to evade the immune system and promote tumor metastasis and progression [50,183,184]. Therefore, careful evaluation is needed when using them clinically. Concurrently, modulation of the estrogen signaling axis continues to advance. A similarly complex picture emerges in the regulation of the sex hormone axis. A phase III trial enrolling patients with ER-positive/HER2-negative advanced breast cancer showed that the novel oral estrogen receptor degrader vepdegestrant significantly extended progression-free survival in the *ESR1*-mutant subgroup but did not achieve statistical significance in the overall population [185]. These findings suggest that the efficacy of sex hormone signaling modulation is highly dependent on the molecular subtype of the tumor, making precise companion diagnostics essential for realizing the benefits of such interventions. Furthermore, sex differences in immune responses associated with sex hormones also suggest that future treatment strategies may need to incorporate a “sex perspective” to optimize individualized immunotherapy combinations through the modulation of sex hormone signaling [186].

#### 4.3.4. Non-Pharmacological Behavioral Interventions

Non-pharmacological behavioral interventions such as acupuncture, exercise, and mindfulness training have emerged as important complements to host neural-targeted therapies. Foundational research on the neuroanatomy of acupuncture has revealed that electroacupuncture stimulation at specific acupoints drives distinct autonomic pathways in a somatotopic and intensity-dependent manner [187]. Based on this principle, a randomized controlled trial demonstrated that adding true electroacupuncture to standard triple antiemetic therapy significantly improved the complete protection rate for chemotherapy-induced nausea and vomiting in breast cancer patients (52.9% vs. 34.5%) [188], demonstrating its clear value in managing treatment-related toxicities. In terms of improving long-term prognosis, the potential of exercise interventions is increasingly evident. A landmark challenge trial showed that a 3-year structured exercise intervention significantly improved disease-free survival and overall survival in patients with stage II-III colorectal cancer after resection [189], elevating exercise from supportive care to an evidence-based treatment capable of altering disease trajectory. For chemotherapy-related cognitive impairment, exercise modalities such as aerobic dance are being systematically investigated to assess their effects on improving cognition and alleviating fatigue [190]. Additionally, mindfulness interventions, as a behavioral therapy for regulating psychological stress, have been shown in a meta-analysis to significantly reduce anxiety, depression, and fatigue symptoms in patients with lung cancer [191].

## 5. Perspectives and Future Directions

Integrating host–tumor metabolic interactions into the study of cancer therapy resistance has reshaped our understanding of treatment failure, shifting the focus beyond tumor cell-autonomous mechanisms. Despite substantial progress, several bottlenecks remain. This section outlines core challenges, emerging technological opportunities, and translational pathways to guide future research.

### 5.1. Current Challenges

Host–tumor metabolic interactions are inherently heterogeneous and context-dependent. The impact of host factors—aging, diet, and psychological stress—on treatment resistance varies with cancer type, genetic background, sex, and treatment modality, making it difficult to establish a unified theoretical framework. A second challenge lies in the temporal and spatial mismatch between host metabolic dynamics and tumor metabolic adaptation. Most studies still rely on static, single-time-point analyses, which fail to capture the bidirectional evolution of these interactions over time and across different tissue compartments. A third barrier is the translational gap between preclinical models and clinical reality. Traditional animal models often do not recapitulate the complex host backgrounds—such as aging, nutritional imbalances, or chronic stress—that characterize human cancer patients. This disconnect has contributed to the failure of many promising intervention strategies in clinical trials.

### 5.2. Emerging Technologies

Spatiotemporally resolved multi-omics technologies offer powerful tools to address these challenges. Spatial transcriptomics, spatial metabolomics, and multiplex imaging enable in situ analysis of metabolic interactions among senescent cells, immune cells, and tumor cells within native tissue architecture. When integrated with single-cell-resolution multi-omics data, these approaches allow reconstruction of spatiotemporal dynamic maps of cellular metabolic networks, facilitating precise identification of resistance-driving metabolic nodes.

Artificial intelligence and machine learning are also reshaping the research landscape. By integrating multidimensional data from large clinical cohorts—including metabolomics, genomics, imaging, and real-world clinical data—deep learning models can identify host metabolic signatures that predict treatment response and enable precise patient stratification. Moreover, AI-assisted drug repurposing can systematically screen approved drugs (e.g., beta-blockers, glucocorticoid receptor antagonists) for synergistic potential, substantially accelerating clinical translation.

### 5.3. Clinical Translation Strategies

Safely, effectively, and feasibly integrating host metabolic modulation into existing cancer treatment regimens remains a central challenge. A key principle is recognizing that interventions such as dietary modifications and behavioral strategies serve an adjuvant role—sensitizing tumors, improving treatment tolerability, and alleviating toxicity—rather than replacing standard anticancer therapies. Future efforts should focus on establishing integrated implementation pathways that combine host metabolic interventions with standard treatments synergistically. This requires, first, prospective clinical studies to define appropriate patient populations, intervention timing, dosage, duration, and safety profiles for strategies ranging from dietary interventions to metabolism-targeting drugs. Such studies will lay the groundwork for standardized, scalable clinical guidelines. Second, multidisciplinary collaborative care models—integrating oncology, nutrition, endocrinology, and psychiatry—are needed to enable dynamic monitoring, precise stratification, and individualized regulation of patient metabolic status. Concurrently, improving efficacy prediction and prognostic assessment systems based on host metabolic characteristics will be essential for guiding clinical decision-making. Ultimately, translating the theory of host–tumor metabolic interactions into clinical practice will advance metabolic modulation from a research concept to a tangible component of precision oncology.

## Data Availability

No new data were created or analyzed in this study.

## References

[1] Parida P.K., Malladi S. (2025). Metabolic adaptations of brain metastasis. Nat. Rev. Cancer.

[2] Martínez-Reyes I., Chandel N.S. (2021). Cancer metabolism: Looking forward. Nat. Rev. Cancer.

[3] De Martino M., Rathmell J.C., Galluzzi L., Vanpouille-Box C. (2024). Cancer cell metabolism and antitumour immunity. Nat. Rev. Immunol..

[4] Scolaro T., Manco M., Pecqueux M., Amorim R., Trotta R., Van Acker H.H., Van Haele M., Shirgaonkar N., Naulaerts S., Daniluk J. (2024). Nucleotide metabolism in cancer cells fuels a UDP-driven macrophage cross-talk, promoting immunosuppression and immunotherapy resistance. Nat. Cancer.

[5] Vitale I., Manic G., Coussens L.M., Kroemer G., Galluzzi L. (2019). Macrophages and Metabolism in the Tumor Microenvironment. Cell Metab..

[6] Aliazis K., Christofides A., Shah R., Yeo Y.Y., Jiang S., Charest A., Boussiotis V.A. (2025). The tumor microenvironment’s role in the response to immune checkpoint blockade. Nat. Cancer.

[7] Murase Y., Nanjo S., Ueda T., Liu Y., Nomura S., Arai S., Terada N., Koba H., Tambo Y., Yano S. (2025). Mechanisms of resistance to antibody-drug conjugates in cancers. Respir. Investig..

[8] Swanton C., Bernard E., Abbosh C., André F., Auwerx J., Balmain A., Bar-Sagi D., Bernards R., Bullman S., DeGregori J. (2024). Embracing cancer complexity: Hallmarks of systemic disease. Cell.

[9] Altea-Manzano P., Decker-Farrell A., Janowitz T., Erez A. (2025). Metabolic interplays between the tumour and the host shape the tumour macroenvironment. Nat. Rev. Cancer.

[10] Febbraio M.A., Pedersen B.K. (2026). Exercise as a therapeutic intervention for long-lasting and chronic diseases. Cell Metab..

[11] Bass J. (2024). Interorgan rhythmicity as a feature of healthful metabolism. Cell Metab..

[12] Goncalves R.L.S., Wang Z.B., Riveros J.K., Parlakgül G., Inouye K.E., Lee G.Y., Fu X., Saksi J., Rosique C., Hui S.T. (2025). CoQ imbalance drives reverse electron transport to disrupt liver metabolism. Nature.

[13] Wagle N.S., Nogueira L., Devasia T.P., Mariotto A.B., Yabroff K.R., Islami F., Jemal A., Alteri R., Ganz P.A., Siegel R.L. (2025). Cancer treatment and survivorship statistics, 2025. CA Cancer J. Clin..

[14] Baden D., Wolgast N., Altrock P.M., Steinhäuser S., Voran J., Beder T., Hecht M., Baden C., Bastian L., Ronckers C. (2025). Epidemiology, survival, and treatment of acute myeloid and lymphoblastic leukaemia in Germany: A nationwide population-based registry analysis. Lancet Reg. Health Eur..

[15] Caruso G., Weroha S.J., Cliby W. (2025). Ovarian Cancer: A Review. JAMA.

[16] Zhou T., Yan J., Zhang Y., Shao F., Xu J., Hu T., Mao G., Shi S., Zhang Y., Hao J. (2026). Senescence as the Next Frontier in Cancer Research: Navigating the Rising Tide of Geriatric Oncology. Cancer Discov..

[17] Gomes A.P., Ilter D., Low V., Endress J.E., Fernández-García J., Rosenzweig A., Schild T., Broekaert D., Ahmed A., Planque M. (2020). Age-induced accumulation of methylmalonic acid promotes tumour progression. Nature.

[18] González-Gualda E., Reinius M.A.V., Macias D., Morsli S., Ge J., Olan I., Martín J.E., Ou H.-L., Hartono M., Puerto-Camacho M.P. (2026). Treatment resistance to platinum-based chemotherapy in lung and ovarian cancer is driven by a targetable TGFβ senescent secretome. Nat. Aging.

[19] Alicea G.M., Rebecca V.W., Goldman A.R., Fane M.E., Douglass S.M., Behera R., Webster M.R., Kugel C.H., Ecker B.L., Caino M.C. (2020). Changes in Aged Fibroblast Lipid Metabolism Induce Age-Dependent Melanoma Cell Resistance to Targeted Therapy via the Fatty Acid Transporter FATP2. Cancer Discov..

[20] Fane M.E., Chhabra Y., Alicea G.M., Maranto D.A., Douglass S.M., Webster M.R., Rebecca V.W., Marino G.E., Almeida F., Ecker B.L. (2022). Stromal changes in the aged lung induce an emergence from melanoma dormancy. Nature.

[21] Chhabra Y., Fane M.E., Pramod S., Hüser L., Zabransky D.J., Wang V., Dixit A., Zhao R., Kumah E., Brezka M.L. (2024). Sex-dependent effects in the aged melanoma tumor microenvironment influence invasion and resistance to targeted therapy. Cell.

[22] Hope H.C., de Sostoa J., Ginefra P., Andreatta M., Chiang Y.-H., Ronet C., Pich-Bavastro C., Corria Osorio J., Kuonen F., Auwerx J. (2025). Age-associated nicotinamide adenine dinucleotide decline drives CAR-T cell failure. Nat. Cancer.

[23] Wang F., Bao R., Xu S., Li W., Huang H., Li R., Ding X., Zhang Y., Yu X., Han Q. (2025). An age-related decrease in leptin contributes to CD8^+^ T cell aging in the tumor microenvironment. Cell Rep. Med..

[24] Parsons A., Sauras Colón E., Manjunath M., Zhang H., Chen J., Spasic M., Koca B., Binboga Kurt B., Freedman R.A., Mittendorf E.A. (2025). Cell populations in human breast cancers are molecularly and biologically distinct with age. Nat. Aging.

[25] Sceneay J., Goreczny G.J., Wilson K., Morrow S., DeCristo M.J., Ubellacker J.M., Qin Y., Laszewski T., Stover D.G., Barrera V. (2019). Interferon Signaling Is Diminished with Age and Is Associated with Immune Checkpoint Blockade Efficacy in Triple-Negative Breast Cancer. Cancer Discov..

[26] Chen M.-L., He Y., Dong X.-H., Liu H.-F., Yan Z.-X., Lu X.-L., Miao Q.-Q., Zhao Q.-N., Zhang H., Luo L. (2025). Ketogenic diet inhibits glioma progression by promoting gut microbiota-derived butyrate production. Cancer Cell.

[27] Liu S., Guruprasad P., Ramasubramanian R., Madhu B., Paruzzo L., Han K., Kelly A., Shestov A., Xu H.N., Carturan A. (2026). β-hydroxybutyrate enhances the metabolic fitness of CAR T cells in cancer. Cell.

[28] Yang L., TeSlaa T., Ng S., Nofal M., Wang L., Lan T., Zeng X., Cowan A., McBride M., Lu W. (2022). Ketogenic diet and chemotherapy combine to disrupt pancreatic cancer metabolism and growth. Med.

[29] Roichman A., Zuo Q., Hwang S., Lu W., Cordova R.A., MacArthur M.R., Boyer J.A., Mitchell S.J., Powers J., Koval S.A. (2025). Microbiome metabolism of dietary phytochemicals controls the anticancer activity of PI3K inhibitors. Cell.

[30] Oswald B.M., DeCamp L.M., Longo J., Dahabieh M.S., Bunda N., Johnson B.K., Watson M.J., Ma S., Preston S.E.J., Sheldon R.D. (2025). Dietary restriction reprograms CD8^+^ T cell fate to enhance anti-tumour immunity and immunotherapy responses. Nat. Metab..

[31] Ajona D., Ortiz-Espinosa S., Lozano T., Exposito F., Calvo A., Valencia K., Redrado M., Remírez A., Lecanda F., Alignani D. (2020). Short-term starvation reduces IGF-1 levels to sensitize lung tumors to PD-1 immune checkpoint blockade. Nat. Cancer.

[32] Delconte R.B., Owyong M., Santosa E.K., Srpan K., Sheppard S., McGuire T.J., Abbasi A., Diaz-Salazar C., Chun J., Rogatsky I. (2024). Fasting reshapes tissue-specific niches to improve NK cell-mediated anti-tumor immunity. Immunity.

[33] Padrão N., Severson T.M., Gregoricchio S., Guijarro A., Lutz C., Taranto D., Hutten S., Ligorio F., Persia A., Roest M. (2025). Fasting boosts breast cancer therapy efficacy via glucocorticoid activation. Nature.

[34] Tintelnot J., Xu Y., Lesker T.R., Schönlein M., Konczalla L., Giannou A.D., Pelczar P., Kylies D., Puelles V.G., Bielecka A.A. (2023). Microbiota-derived 3-IAA influences chemotherapy efficacy in pancreatic cancer. Nature.

[35] Bender M.J., McPherson A.C., Phelps C.M., Pandey S.P., Laughlin C.R., Shapira J.H., Medina Sanchez L., Rana M., Richie T.G., Mims T.S. (2023). Dietary tryptophan metabolite released by intratumoral Lactobacillus reuteri facilitates immune checkpoint inhibitor treatment. Cell.

[36] De Juan A., Coillard A., Cros A., Rigamonti A., Alaoui L., Sampaio J.L., Monot N., Balvay A., Foussier A., Rieux-Laucat M. (2025). Physiological activation of Aryl hydrocarbon receptor by food-derived ligands is essential for the efficacy of anti-PD1 therapy. Nat. Commun..

[37] Kanarek N., Keys H.R., Cantor J.R., Lewis C.A., Chan S.H., Kunchok T., Abu-Remaileh M., Freinkman E., Schweitzer L.D., Sabatini D.M. (2018). Histidine catabolism is a major determinant of methotrexate sensitivity. Nature.

[38] Qian Z.Y., Deng Y., Xi M., Zhang X.Y., Sun H., Mao M., Luo X.J., Li M., Zhang Q., Chen B. (2026). Longitudinal Plasma Metabolomics Guides Dynamic Risk Assessment and Dietary Modulation for Esophageal Squamous Cell Cancer Chemoimmunotherapy. Cancer Discov..

[39] Bolte L.A., Lee K.A., Björk J.R., Leeming E.R., Campmans-Kuijpers M.J.E., de Haan J.J., Vila A.V., Maltez-Thomas A., Segata N., Board R. (2023). Association of a Mediterranean Diet with Outcomes for Patients Treated with Immune Checkpoint Blockade for Advanced Melanoma. JAMA Oncol..

[40] Kok D.E., Arron M.N.N., Huibregtse T., Kruyt F.M., Bac D.J., van Halteren H.K., Kouwenhoven E.A., Wesselink E., Winkels R.M., van Zutphen M. (2021). Association of Habitual Preoperative Dietary Fiber Intake with Complications After Colorectal Cancer Surgery. JAMA Surg..

[41] Lanser D.A.C., de Leeuw S.P., Oomen-de Hoop E., de Bruijn P., Paats M.S., Dumoulin D.W., Koolen S.L.W., Dingemans A.-M.C., Mathijssen R.H.J., Veerman G.D.M. (2023). Influence of Food with Different Fat Concentrations on Alectinib Exposure: A Randomized Crossover Pharmacokinetic Trial. J. Natl. Compr. Cancer Netw..

[42] Spencer C.N., McQuade J.L., Gopalakrishnan V., McCulloch J.A., Vetizou M., Cogdill A.P., Khan M.A.W., Zhang X., White M.G., Peterson C.B. (2021). Dietary fiber and probiotics influence the gut microbiome and melanoma immunotherapy response. Science.

[43] Zhou P., Chang W.-y., Gong D.-a., Xia J., Chen W., Huang L.-y., Liu R., Liu Y., Chen C., Wang K. (2023). High dietary fructose promotes hepatocellular carcinoma progression by enhancing O-GlcNAcylation via microbiota-derived acetate. Cell Metab..

[44] Niu X., Wu T., Zeng L., Wang F., Lv W., Zhang L., Zhou F. (2025). Chronic stress in cancer development and progression. Sci. Bull..

[45] Eckerling A., Ricon-Becker I., Sorski L., Sandbank E., Ben-Eliyahu S. (2021). Stress and cancer: Mechanisms, significance and future directions. Nat. Rev. Cancer.

[46] Khan A., Song M., Dong Z. (2025). Chronic stress: A fourth etiology in tumorigenesis?. Mol. Cancer.

[47] Ma Y., Kroemer G. (2023). The cancer-immune dialogue in the context of stress. Nat. Rev. Immunol..

[48] Nilsson M.B., Sun H., Diao L., Tong P., Liu D., Li L., Fan Y., Poteete A., Lim S.-O., Howells K. (2017). Stress hormones promote EGFR inhibitor resistance in NSCLC: Implications for combinations with β-blockers. Sci. Transl. Med..

[49] Yang H., Xia L., Chen J., Zhang S., Martin V., Li Q., Lin S., Chen J., Calmette J., Lu M. (2019). Stress–glucocorticoid–TSC22D3 axis compromises therapy-induced antitumor immunity. Nat. Med..

[50] Cassandras M., Sanchez X., Hsu L., Huang Y., Getzler A.J., Ganguly D., Baldominos P., Codinachs I., Chuong J., Martin E.E. (2026). A glucocorticoid–FAS axis controls immune evasion during metastatic seeding. Nature.

[51] Zeng Y., Hu C.-H., Li Y.-Z., Zhou J.-S., Wang S.-X., Liu M.-D., Qiu Z.-H., Deng C., Ma F., Xia C.-F. (2024). Association between pretreatment emotional distress and immune checkpoint inhibitor response in non-small-cell lung cancer. Nat. Med..

[52] Chen M., Qiao G., Hylander B.L., Mohammadpour H., Wang X.-Y., Subjeck J.R., Singh A.K., Repasky E.A. (2020). Adrenergic stress constrains the development of anti-tumor immunity and abscopal responses following local radiation. Nat. Commun..

[53] Huang C., Tao H., Zhou Y., Wu Q., Li M., Liu A., Zhu T., Yu C., Li P., Huang S. (2026). Pregnenolone promotes immune evasion through blocking endogenous retrovirus expression. Cell Metab..

[54] Guan Y., Yao W., Yu H., Feng Y., Zhao Y., Zhan X., Wang Y. (2023). Chronic stress promotes colorectal cancer progression by enhancing glycolysis through β2-AR/CREB1 signal pathway. Int. J. Biol. Sci..

[55] Luo M., Luo W. (2026). Aminopeptidase N: The glucocorticoid gateway linking chronic stress to ferroptosis resistance in liver cancer. J. Clin. Investig..

[56] Sun R., Jiao D., Yuan W., Wang H., Ren L., Fu Z., Zhang J., Yue X., Wu Z., Li C. (2026). Chronic stress drives liver cancer by impairing the hepatic kynurenine pathway and immune surveillance. Nat. Metab..

[57] Li J., Lu J., Zheng C., Huang X., Li H., Mai Q., Chen S., Zhou Z., Zhu J., Yu T. (2026). Serotonin-licensed macrophages potentiate chemoresistance via inositol metabolic crosstalk in ovarian cancer. Cell Metab..

[58] Chen Z., Zhou Y., Xue C., Zeng L., Deng S., Xu Z., Li M., Zhao H., He X., Liu S. (2025). Psychological stress-induced ALKBH5 deficiency promotes tumour innervation and pancreatic cancer via extracellular vesicle transfer of RNA. Nat. Cell Biol..

[59] Zhang Y., Guo Y., Liu Z., Sun Y., Yang X., Chen M., Feng G., Lin C., Wang Y., Zhang Z. (2025). Cancer cells co-opt an inter-organ neuroimmune circuit to escape immune surveillance. Cell.

[60] Bucsek M.J., Qiao G., MacDonald C.R., Giridharan T., Evans L., Niedzwecki B., Liu H., Kokolus K.M., Eng J.W.L., Messmer M.N. (2017). β-Adrenergic Signaling in Mice Housed at Standard Temperatures Suppresses an Effector Phenotype in CD8+ T Cells and Undermines Checkpoint Inhibitor Therapy. Cancer Res..

[61] Cui B., Luo H., He B., Liu X., Lv D., Zhang X., Su K., Zheng S., Lu J., Wang C. (2025). Gut dysbiosis conveys psychological stress to activate LRP5/β-catenin pathway promoting cancer stemness. Signal Transduct. Target. Ther..

[62] Hoover G., Gilbert S., Curley O., Obellianne C., Lin M.T., Hixson W., Pierce T.W., Andrews J.F., Alexeyev M.F., Ding Y. (2025). Nerve-to-cancer transfer of mitochondria during cancer metastasis. Nature.

[63] Peinado P., Stazi M., Ballabio C., Margineanu M.-B., Li Z., Colón C.I., Hsieh M.-S., Pal Choudhuri S., Stastny V., Hamilton S. (2025). Intrinsic electrical activity drives small-cell lung cancer progression. Nature.

[64] Thiel V., Renders S., Panten J., Dross N., Bauer K., Azorin D., Henriques V., Vogel V., Klein C., Leppä A.-M. (2025). Characterization of single neurons reprogrammed by pancreatic cancer. Nature.

[65] Winkler F., Venkatesh H.S., Amit M., Batchelor T., Demir I.E., Deneen B., Gutmann D.H., Hervey-Jumper S., Kuner T., Mabbott D. (2023). Cancer neuroscience: State of the field, emerging directions. Cell.

[66] Zhang Y., Zhuang W., Zhu H., Meng F., Chen M., Guo Y., Fan J., Sun Y., Ji T. (2026). Neuroimmunometabolic co-evolution in the tumor micro- and macroenvironment. Cell Metab..

[67] Pich O., Ward S., Rowan A., Naceur-Lombardelli C., Shutkever O., Martinez-Ruiz C., Harries S., Hessey S., Naidu B., Brenton J.D. (2025). Somatic evolution following cancer treatment in normal tissue. Nature.

[68] Smitherman A.B., Wood W.A., Mitin N., Ayer Miller V.L., Deal A.M., Davis I.J., Blatt J., Gold S.H., Muss H.B. (2020). Accelerated aging among childhood, adolescent, and young adult cancer survivors is evidenced by increased expression of p16INK4a and frailty. Cancer.

[69] Williams A.M., Phillips N.S., Dong Q., Ehrhardt M.J., Gilmore N., Loh K.P., Meng X., Ness K.K., Hudson M.M., Robison L.L. (2025). Epigenetic age acceleration, telomere length, and neurocognitive function in long-term survivors of childhood cancer. Nat. Commun..

[70] Krawczuk-Rybak M., Latoch E. (2019). Risk factors for premature aging in childhood cancer survivors. Dev. Period Med..

[71] Zhang X., Nie Z., Wang S., Ma Y., Han D., Hu T., Liu L., Men L., Zhang T., Wu X. (2025). Arachidonic acid triggers spermidine synthase secretion from primary tumor to induce skeletal muscle weakness upon irradiation. Cell Metab..

[72] Fletcher-Sananikone E., Kanji S., Tomimatsu N., Di Cristofaro L.F.M., Kollipara R.K., Saha D., Floyd J.R., Sung P., Hromas R., Burns T.C. (2021). Elimination of Radiation-Induced Senescence in the Brain Tumor Microenvironment Attenuates Glioblastoma Recurrence. Cancer Res..

[73] Lin K., Wei L., Wang R., Li L., Song S., Wang F., He M., Pu W., Wang J., Wazir J. (2025). Disrupted methionine cycle triggers muscle atrophy in cancer cachexia through epigenetic regulation of REDD1. Cell Metab..

[74] Ma F., Liu X., Zhang Y., Tao Y., Zhao L., Abusalamah H., Huffman C., Harbison R.A., Puram S.V., Wang Y. (2025). Tumor extracellular vesicle–derived PD-L1 promotes T cell senescence through lipid metabolism reprogramming. Sci. Transl. Med..

[75] Dai D., Pei Y., Zhu B., Wang D., Pei S., Huang H., Zhu Q., Deng X., Ye J., Xu J. (2024). Chemoradiotherapy-induced ACKR2+ tumor cells drive CD8+ T cell senescence and cervical cancer recurrence. Cell Rep. Med..

[76] Nicolas A.M., Pesic M., Engel E., Ziegler P.K., Diefenhardt M., Kennel K.B., Buettner F., Conche C., Petrocelli V., Elwakeel E. (2022). Inflammatory fibroblasts mediate resistance to neoadjuvant therapy in rectal cancer. Cancer Cell.

[77] Yousefzadeh M.J., Flores R.R., Zhu Y., Schmiechen Z.C., Brooks R.W., Trussoni C.E., Cui Y., Angelini L., Lee K.-A., McGowan S.J. (2021). An aged immune system drives senescence and ageing of solid organs. Nature.

[78] Hesterberg R.S., Davis J.T., Handoo K.J., Elmarsafawi A.G., Augello A.C., Cheng C.-H., Atkins R., Lee D.H., Yang C., Yao J. (2025). Lymphoma accelerates T cell and tissue aging. Cancer Cell.

[79] Liu L., Wang Q., Chen M., Zhou H., Li X., Yuan Z., Hu Y., Wang C., Zhang X., Hu S. (2025). Cancer-cell-secreted DDAH1 induces TGF-β1/Smad3 signaling pathway to promote fibrosis and aging in lung. Nat. Aging.

[80] Demaria M., O’Leary M.N., Chang J., Shao L., Liu S., Alimirah F., Koenig K., Le C., Mitin N., Deal A.M. (2017). Cellular Senescence Promotes Adverse Effects of Chemotherapy and Cancer Relapse. Cancer Discov..

[81] Karschnia P., Nelson T.A., Dietrich J. (2025). Mechanisms and treatment of cancer therapy-induced peripheral and central neurotoxicity. Nat. Rev. Cancer.

[82] Heinrich M., Hofmann L., Baurecht H., Kreuzer P.M., Knüttel H., Leitzmann M.F., Seliger C. (2022). Suicide risk and mortality among patients with cancer. Nat. Med..

[83] Miller N.E., Pentti J., Steptoe A., Kivimaki M., Lally P., Frank P., Fisher A. (2025). Mediators of the association between psychological distress and mortality in people diagnosed with cancer. Nat. Commun..

[84] Janelsins M.C., Heckler C.E., Peppone L.J., Kamen C., Mustian K.M., Mohile S.G., Magnuson A., Kleckner I.R., Guido J.J., Young K.L. (2017). Cognitive Complaints in Survivors of Breast Cancer After Chemotherapy Compared with Age-Matched Controls: An Analysis From a Nationwide, Multicenter, Prospective Longitudinal Study. J. Clin. Oncol..

[85] Kang I.M., Forschmiedt J.K., Loch M.M., Lew D.L., Barlow W.E., Gralow J.R., Meric-Bernstam F., Albain K.S., Hayes D.F., Lin N.U. (2026). Cognitive Impairment and Chemoendocrine vs Endocrine Therapy in Pre- and Postmenopausal Women. JAMA Oncol..

[86] Sleurs C., Zegers C.M.L., Ribeiro M.F., van Elmpt W., Dijkstra J., Postma A.A., De Roeck L., Gehring K., De Baene W., Sitskoorn M.M. (2025). Radiotherapy-induced neurocognitive decline among adult intracranial tumor patients: A voxel-based approach. Neuro-Oncology.

[87] Bennett C.L., Focosi D., Socal M.P., Bian J.C., Nabhan C., Hrushesky W.J., Bennett A.C., Schoen M.W., Berger J.R., Armitage J.O. (2021). Progressive multifocal leukoencephalopathy in patients treated with rituximab: A 20-year review from the Southern Network on Adverse Reactions. Lancet Haematol..

[88] Gibson E.M., Nagaraja S., Ocampo A., Tam L.T., Wood L.S., Pallegar P.N., Greene J.J., Geraghty A.C., Goldstein A.K., Ni L. (2019). Methotrexate Chemotherapy Induces Persistent Tri-glial Dysregulation that Underlies Chemotherapy-Related Cognitive Impairment. Cell.

[89] Groves T.R., Farris R., Anderson J.E., Alexander T.C., Kiffer F., Carter G., Wang J., Boerma M., Allen A.R. (2017). 5-Fluorouracil chemotherapy upregulates cytokines and alters hippocampal dendritic complexity in aged mice. Behav. Brain Res..

[90] Liu Y., Reiken S., Dridi H., Yuan Q., Mohammad K.S., Trivedi T., Miotto M.C., Wedderburn-Pugh K., Sittenfeld L., Kerley Y. (2023). Targeting ryanodine receptor type 2 to mitigate chemotherapy-induced neurocognitive impairments in mice. Sci. Transl. Med..

[91] Geraghty A.C., Acosta-Alvarez L., Rotiroti M.C., Dutton S., O’Dea M.R., Kim W., Trivedi V., Mancusi R., Shamardani K., Malacon K. (2025). Immunotherapy-related cognitive impairment after CAR T cell therapy in mice. Cell.

[92] Berger S.C., Fehse B., Akyüz N., Geffken M., Wolschke C., Janson D., Gagelmann N., Luther M., Wichmann D., Frenzel C. (2022). Molecular monitoring of T-cell kinetics and migration in severe neurotoxicity after real-world CD19-specific chimeric antigen receptor T cell therapy. Haematologica.

[93] Staedtke V., Bai R.-Y., Kim K., Darvas M., Davila M.L., Riggins G.J., Rothman P.B., Papadopoulos N., Kinzler K.W., Vogelstein B. (2018). Disruption of a self-amplifying catecholamine loop reduces cytokine release syndrome. Nature.

[94] Yang X., Wang X., Lu W., Ye Q., Li Y., Ma X., Ye Y., Guo X., Chen G., Xin W. (2026). *METTL14* integrates tumor-derived SAM to drive parabrachial epigenetic rewiring in pancreatic cancer. Neuron.

[95] Zhu X., Shen J., Feng S., Huang C., Wang H., Huo F., Liu H. (2023). Akkermansia muciniphila, which is enriched in the gut microbiota by metformin, improves cognitive function in aged mice by reducing the proinflammatory cytokine interleukin-6. Microbiome.

[96] Kumazawa T., Xu Y., Wang Y., Lee J.-W., O’Brien T.C., Ho C.-K., Cetinbas M., Weiner A., Hochedlinger K., Sadreyev R.I. (2026). Metformin inhibits nuclear egress of chromatin fragments in senescence and aging. Nat. Aging.

[97] Zhang J., Cai W., Liu D., Zheng N., Wang Y., Qiu F., Zheng H., Gan H., Huang Y., Zhou Y. (2025). Effect of henagliflozin on aging biomarkers in patients with type 2 diabetes: A multicenter, randomized, double-blind, placebo-controlled study. Cell Rep. Med..

[98] Gozdecka M., Dudek M., Wen S., Gu M., Stopforth R.J., Rak J., Damaskou A., Grice G.L., McLoughlin M.A., Bond L. (2025). Mitochondrial metabolism sustains DNMT3A-R882-mutant clonal haematopoiesis. Nature.

[99] Hosseini M., Voisin V., Chegini A., Varesi A., Cathelin S., Ayyathan D.M., Liu A.C.H., Yang Y., Wang V., Maher A. (2025). Metformin reduces the competitive advantage of Dnmt3aR878H HSPCs. Nature.

[100] Elgendy M., Cirò M., Hosseini A., Weiszmann J., Mazzarella L., Ferrari E., Cazzoli R., Curigliano G., DeCensi A., Bonanni B. (2019). Combination of Hypoglycemia and Metformin Impairs Tumor Metabolic Plasticity and Growth by Modulating the PP2A-GSK3β-MCL-1 Axis. Cancer Cell.

[101] Eilenberg W., Klopf J., Sotir A., Scheuba A., Domenig C., Loewe C., Dalman R., Wanhainen A., Sakalihasan N., Ristl R. (2025). Editor’s Choice—Metformin to Inhibit Progression of Abdominal Aortic Aneurysm: A Randomised, Placebo Controlled Clinical Trial. Eur. J. Vasc. Endovasc. Surg..

[102] Cheng C., Geng F., Li Z., Zhong Y., Wang H., Cheng X., Zhao Y., Mo X., Horbinski C., Duan W. (2022). Ammonia stimulates SCAP/Insig dissociation and SREBP-1 activation to promote lipogenesis and tumour growth. Nat. Metab..

[103] Liu J., Shen Y., Liu J., Xu D., Chang C.-Y., Wang J., Zhou J., Haffty B.G., Zhang L., Bargonetti J. (2025). Lipogenic enzyme FASN promotes mutant p53 accumulation and gain-of-function through palmitoylation. Nat. Commun..

[104] Sun J., Wang J.-M., Zhang Q., Lin W.-Y., Tang L., Lu S.-Y., Li B.-Q., Du Z.-X., Wang H.-Q. (2025). The simultaneous targeted Inhibition of *ISG15* and HMGCR disrupts cancer stemness through metabolic collapse and induces synthetic lethality in pancreatic ductal adenocarcinoma. J. Exp. Clin. Cancer Res..

[105] Mittal S., Nenwani M., Pulikkal Kadamberi I., Kumar S., Animasahun O., George J., Tsaih S.W., Gupta P., Singh M., Geethadevi A. (2025). eIF4E Enriched Extracellular Vesicles Induce Immunosuppressive Macrophages through HMGCR-Mediated Metabolic Rewiring. Adv. Sci..

[106] Zhong Y., Geng F., Su H., Mazik L., Yin X., Pan M., Li N., Chiang C.Y., Tonniges J.R., Mo X. (2026). SREBP-1 increases glucose uptake to promote tumor resistance to lysosome inhibition. Sci. Transl. Med..

[107] Yu Z., Li Q., Ding M., Ping X., Gu W., Yi Q., Dai J., Tian R., Pan Z., Zheng L. (2025). Exercise improves aging-induced cardiac dysfunction and prolongs lifespan via Hmgcr. Life Sci..

[108] Gong Z., Zhu J., Chen J., Feng F., Zhang H., Zhang Z., Song C., Liang K., Yang S., Fan S. (2023). CircRREB1 mediates lipid metabolism related senescent phenotypes in chondrocytes through FASN post-translational modifications. Nat. Commun..

[109] Li Y., Tang S., Wang H., Zhu H., Lu Y., Zhang Y., Guo S., He J., Li Y., Zhang Y. (2025). A pancreatic cancer organoid biobank links multi-omics signatures to therapeutic response and clinical evaluation of statin combination therapy. Cell Stem Cell.

[110] Lao M., Zhang X., Li Z., Sun K., Yang H., Wang S., He L., Chen Y., Zhang H., Shi J. (2025). Lipid metabolism reprograming by SREBP1-PCSK9 targeting sensitizes pancreatic cancer to immunochemotherapy. Cancer Commun..

[111] Banerjee M., Zaytseva Y.Y., Reusch E.M., Napier D.L., Hasani S., Rychahou P., Izumi T., Cheek D.A., Li J., Flight R.M. (2026). FASN Inhibition Enhances the Efficacy of Chemotherapy in Colorectal Cancer by Inhibiting the DNA Damage Response. Cancer Res..

[112] Kosakamoto H., Obata F., Kuraishi J., Aikawa H., Okada R., Johnstone J.N., Onuma T., Piper M.D.W., Miura M. (2023). Early-adult methionine restriction reduces methionine sulfoxide and extends lifespan in *Drosophila*. Nat. Commun..

[113] Rajabian N., Ikhapoh I., Shahini S., Choudhury D., Thiyagarajan R., Shahini A., Kulczyk J., Breed K., Saha S., Mohamed M.A. (2023). Methionine adenosyltransferase2A inhibition restores metabolism to improve regenerative capacity and strength of aged skeletal muscle. Nat. Commun..

[114] Hernández-Arciga U., Stamenkovic C., Yadav S., Nicoletti C., Albalawy W.N., Al Hammood F., Gonzalez T.F., Naikwadi M.U., Graham A., Smarz C. (2025). Dietary methionine restriction started late in life promotes healthy aging in a sex-specific manner. Sci. Adv..

[115] Bian Y., Li W., Kremer D.M., Sajjakulnukit P., Li S., Crespo J., Nwosu Z.C., Zhang L., Czerwonka A., Pawłowska A. (2020). Cancer *SLC43A2* alters T cell methionine metabolism and histone methylation. Nature.

[116] Gao X., Sanderson S.M., Dai Z., Reid M.A., Cooper D.E., Lu M., Richie J.P., Ciccarella A., Calcagnotto A., Mikhael P.G. (2019). Dietary methionine influences therapy in mouse cancer models and alters human metabolism. Nature.

[117] Xue Y., Lu F., Chang Z., Li J., Gao Y., Zhou J., Luo Y., Lai Y., Cao S., Li X. (2023). Intermittent dietary methionine deprivation facilitates tumoral ferroptosis and synergizes with checkpoint blockade. Nat. Commun..

[118] Mattes M.D., Koturbash I., Leung C.N., Wen S., Jacobson G.M. (2024). A Phase I Trial of a Methionine Restricted Diet with Concurrent Radiation Therapy. Nutr. Cancer.

[119] Chen H., Huang N., Xu W., Yang Y., Wang F., Gong H., Zhou J., Tai H., Zhao T., Zhang J. (2026). Hyperglutaminolysis drives senescence and aging through arginine-mTORC1 axis activation. Signal Transduct. Target. Ther..

[120] Johmura Y., Yamanaka T., Omori S., Wang T.W., Sugiura Y., Matsumoto M., Suzuki N., Kumamoto S., Yamaguchi K., Hatakeyama S. (2021). Senolysis by glutaminolysis inhibition ameliorates various age-associated disorders. Science.

[121] DiNardo C.D., Verma D., Baran N., Bhagat T.D., Skwarska A., Lodi A., Saxena K., Cai T., Su X., Guerra V.A. (2024). Glutaminase inhibition in combination with azacytidine in myelodysplastic syndromes: A phase 1b/2 clinical trial and correlative analyses. Nat. Cancer.

[122] Tannir N.M., Agarwal N., Porta C., Lawrence N.J., Motzer R., McGregor B., Lee R.J., Jain R.K., Davis N., Appleman L.J. (2022). Efficacy and Safety of Telaglenastat Plus Cabozantinib vs Placebo Plus Cabozantinib in Patients with Advanced Renal Cell Carcinoma. JAMA Oncol..

[123] Patel A.A.H., Dzanan J.J., Ali K.X., Eklund E.A., Alvarez S.W., Raj D., Dankis M., Altinönder I., Schwarz M., Le Gal K. (2026). Ageing promotes metastasis via activation of the integrated stress response. Nature.

[124] Abud G.F., De Carvalho F.G., Batitucci G., Travieso S.G., Bueno Junior C.R., Barbosa Junior F., Marchini J.S., de Freitas E.C. (2022). Taurine as a possible antiaging therapy: A controlled clinical trial on taurine antioxidant activity in women ages 55 to 70. Nutrition.

[125] Cao T., Zhang W., Wang Q., Wang C., Ma W., Zhang C., Ge M., Tian M., Yu J., Jiao A. (2024). Cancer SLC6A6-mediated taurine uptake transactivates immune checkpoint genes and induces exhaustion in CD8(+) T cells. Cell.

[126] Fernandez M.E., Bernier M., Price N.L., Camandola S., Aon M.A., Vaughan K., Mattison J.A., Preston J.D., Jones D.P., Tanaka T. (2025). Is taurine an aging biomarker?. Science.

[127] Marcangeli V., Cefis M., Hammad R., Granet J., Leduc-Gaudet J.P., Gaudreau P., Aubertin-Leheudre M., Bélanger M., Robitaille R., Morais J.A. (2025). Experimental Evidence Against Taurine Deficiency as a Driver of Aging in Humans. Aging Cell.

[128] Sharma S., Rodems B.J., Baker C.D., Kaszuba C.M., Franco E.I., Smith B.R., Ito T., Swovick K., Welle K., Zhang Y. (2025). Taurine from tumour niche drives glycolysis to promote leukaemogenesis. Nature.

[129] Singh P., Gollapalli K., Mangiola S., Schranner D., Yusuf M.A., Chamoli M., Shi S.L., Lopes Bastos B., Nair T., Riermeier A. (2023). Taurine deficiency as a driver of aging. Science.

[130] Li S., Hamaya R., Zhu H., Chen B.H., Pereira A.C., Ivey K.L., Rist P.M., Manson J.E., Dong Y., Sesso H.D. (2026). Effects of daily multivitamin–multimineral and cocoa extract supplementation on epigenetic aging clocks in the COSMOS randomized clinical trial. Nat. Med..

[131] Bodeker K.L., Smith B.J., Berg D.J., Chandrasekharan C., Sharif S., Fei N., Vollstedt S., Brown H., Chandler M., Lorack A. (2024). A randomized trial of pharmacological ascorbate, gemcitabine, and nab-paclitaxel for metastatic pancreatic cancer. Redox Biol..

[132] Keum N., Lee D.H., Greenwood D.C., Manson J.E., Giovannucci E. (2019). Vitamin D supplementation and total cancer incidence and mortality: A meta-analysis of randomized controlled trials. Ann. Oncol..

[133] Galus Ł., Michalak M., Lorenz M., Stoińska-Swiniarek R., Tusień Małecka D., Galus A., Kolenda T., Leporowska E., Mackiewicz J. (2023). Vitamin D supplementation increases objective response rate and prolongs progression-free time in patients with advanced melanoma undergoing anti-PD-1 therapy. Cancer.

[134] Grover S., Dougan M., Tyan K., Giobbie-Hurder A., Blum S.M., Ishizuka J., Qazi T., Elias R., Vora K.B., Ruan A.B. (2020). Vitamin D intake is associated with decreased risk of immune checkpoint inhibitor-induced colitis. Cancer.

[135] Roldan Urgoiti G., de Robles P., Tsang R.Y., Willson M., Ghosh S., Faruqi M., Lim G., Loewen S., Nordal R., Cairncross G. (2025). A phase I-II study of niacin in patients with newly diagnosed glioblastoma: Safety and interim phase II analysis. J. Neuro-Oncol..

[136] Cichocki F., Zhang B., Wu C.Y., Chiu E., Day A., O’Connor R.S., Yackoubov D., Simantov R., McKenna D.H., Cao Q. (2023). Nicotinamide enhances natural killer cell function and yields remissions in patients with non-Hodgkin lymphoma. Sci. Transl. Med..

[137] Covarrubias A.J., Perrone R., Grozio A., Verdin E. (2020). NAD+ metabolism and its roles in cellular processes during ageing. Nat. Rev. Mol. Cell Biol..

[138] Yang Q., Chen W., Cong L., Wang M., Li H., Wang H., Luo X., Zhu J., Zeng X., Zhu Z. (2023). NADase CD38 is a key determinant of ovarian aging. Nat. Aging.

[139] Chen Z., Yoo S.-H. (2025). Boosting astrocytic NAD+ against tauopathy. Nat. Aging.

[140] Song W.-S., Shen X., Du K., Ramirez C.B., Park S.H., Cao Y., Le J., Bae H., Kim J., Chun Y. (2025). Nicotinic acid riboside maintains NAD+ homeostasis and ameliorates aging-associated NAD+ decline. Cell Metab..

[141] Shen J., Xu F., Liu T., Ye Y., Xu S. (2025). NAD+ Metabolism-Mediated SURF4-STING Axis Enhances T-Cell Anti-Tumor Effects in the Ovarian Cancer Microenvironment. Cell Death Dis..

[142] Fu T., Jin X., He M., Chen Y.Y., Yang Y.S., Chen L., Zhang H.Y., Fan L., Wu J., Wang Z.H. (2025). Interferon-induced senescent CD8(+) T cells reduce anti-PD1 immunotherapy efficacy in early triple-negative breast cancer. Sci. Transl. Med..

[143] Martens C.R., Denman B.A., Mazzo M.R., Armstrong M.L., Reisdorph N., McQueen M.B., Chonchol M., Seals D.R. (2018). Chronic nicotinamide riboside supplementation is well-tolerated and elevates NAD+ in healthy middle-aged and older adults. Nat. Commun..

[144] Igarashi M., Nakagawa-Nagahama Y., Miura M., Kashiwabara K., Yaku K., Sawada M., Sekine R., Fukamizu Y., Sato T., Sakurai T. (2022). Chronic nicotinamide mononucleotide supplementation elevates blood nicotinamide adenine dinucleotide levels and alters muscle function in healthy older men. npj Aging.

[145] Norheim K.L., Ben Ezra M., Heckenbach I., Andreasson L.M., Eriksen L.L., Dyhre-Petersen N., Damgaard M.V., Berglind M., Pricolo L., Sampson D. (2024). Effect of nicotinamide riboside on airway inflammation in COPD: A randomized, placebo-controlled trial. Nat. Aging.

[146] Al-Habsi M., Chamoto K., Matsumoto K., Nomura N., Zhang B., Sugiura Y., Sonomura K., Maharani A., Nakajima Y., Wu Y. (2022). Spermidine activates mitochondrial trifunctional protein and improves antitumor immunity in mice. Science.

[147] Hofer S.J., Simon A.K., Bergmann M., Eisenberg T., Kroemer G., Madeo F. (2022). Mechanisms of spermidine-induced autophagy and geroprotection. Nat. Aging.

[148] Schroeder S., Hofer S.J., Zimmermann A., Pechlaner R., Dammbrueck C., Pendl T., Marcello G.M., Pogatschnigg V., Bergmann M., Müller M. (2021). Dietary spermidine improves cognitive function. Cell Rep..

[149] Wirth M., Benson G., Schwarz C., Köbe T., Grittner U., Schmitz D., Sigrist S.J., Bohlken J., Stekovic S., Madeo F. (2018). The effect of spermidine on memory performance in older adults at risk for dementia: A randomized controlled trial. Cortex.

[150] Zhang Y., Bai J., Cui Z., Li Y., Gao Q., Miao Y., Xiong B. (2023). Polyamine metabolite spermidine rejuvenates oocyte quality by enhancing mitophagy during female reproductive aging. Nat. Aging.

[151] Zou M., Li D., Yang Y. (2025). The memory- and cognition-facilitating effects of spermidine in aging and aging-related disorders. Ageing Res. Rev..

[152] Vernieri C., Fucà G., Ligorio F., Huber V., Vingiani A., Iannelli F., Raimondi A., Rinchai D., Frigè G., Belfiore A. (2022). Fasting-Mimicking Diet Is Safe and Reshapes Metabolism and Antitumor Immunity in Patients with Cancer. Cancer Discov..

[153] Ligorio F., Vingiani A., Torelli T., Sposetti C., Drufuca L., Iannelli F., Zanenga L., Depretto C., Folli S., Scaperrotta G. (2025). Early downmodulation of tumor glycolysis predicts response to fasting-mimicking diet in triple-negative breast cancer patients. Cell Metab..

[154] Chen S., Hu T., Zhu K., Liu Y., Yang Y., Li X., He J., Cui W., Chi Z., Yu W. (2026). 16-h fasting optimizes cancer immunotherapy in mice and humans. Cell Metab..

[155] Link V.M., Subramanian P., Cheung F., Han K.L., Stacy A., Chi L., Sellers B.A., Koroleva G., Courville A.B., Mistry S. (2024). Differential peripheral immune signatures elicited by vegan versus ketogenic diets in humans. Nat. Med..

[156] Khodabakhshi A., Akbari M.E., Mirzaei H.R., Seyfried T.N., Kalamian M., Davoodi S.H. (2021). Effects of Ketogenic metabolic therapy on patients with breast cancer: A randomized controlled clinical trial. Clin. Nutr..

[157] Cohen C., Fontaine K., Arend R., Soleymani T., Gower B. (2018). Favorable Effects of a Ketogenic Diet on Physical Function, Perceived Energy, and Food Cravings in Women with Ovarian or Endometrial Cancer: A Randomized, Controlled Trial. Nutrients.

[158] Schreck K.C., Hsu F.-C., Berrington A., Henry-Barron B., Vizthum D., Blair L., Kossoff E.H., Easter L., Whitlow C.T., Barker P.B. (2021). Feasibility and Biological Activity of a Ketogenic/Intermittent-Fasting Diet in Patients with Glioma. Neurology.

[159] Fan Y., Hu C., Xie X., Weng Y., Chen C., Wang Z., He X., Jiang D., Huang S., Hu Z. (2024). Effects of diets on risks of cancer and the mediating role of metabolites. Nat. Commun..

[160] Pavlidou E., Papadopoulou S.K., Tolia M., Mentzelou M., Tsoukalas N., Alexatou O., Tsiouda T., Tsourouflis G., Psara E., Bikos V. (2023). Association of Mediterranean Diet Adherence with Disease Progression Characteristics, Lifestyle Factors and Overall Survival in Gastric Cancer Patients. Med. Sci..

[161] Kleckner A.S., Reschke J.E., Kleckner I.R., Magnuson A., Amitrano A.M., Culakova E., Shayne M., Netherby-Winslow C.S., Czap S., Janelsins M.C. (2022). The Effects of a Mediterranean Diet Intervention on Cancer-Related Fatigue for Patients Undergoing Chemotherapy: A Pilot Randomized Controlled Trial. Cancers.

[162] Dizman N., Meza L., Bergerot P., Alcantara M., Dorff T., Lyou Y., Frankel P., Cui Y., Mira V., Llamas M. (2022). Nivolumab plus ipilimumab with or without live bacterial supplementation in metastatic renal cell carcinoma: A randomized phase 1 trial. Nat. Med..

[163] Ebrahimi H., Dizman N., Meza L., Malhotra J., Li X., Dorff T., Frankel P., Llamas-Quitiquit M., Hsu J., Zengin Z.B. (2024). Cabozantinib and nivolumab with or without live bacterial supplementation in metastatic renal cell carcinoma: A randomized phase 1 trial. Nat. Med..

[164] Tomita Y., Goto Y., Sakata S., Imamura K., Minemura A., Oka K., Hayashi A., Jodai T., Akaike K., Anai M. (2022). *Clostridium butyricum* therapy restores the decreased efficacy of immune checkpoint blockade in lung cancer patients receiving proton pump inhibitors. OncoImmunology.

[165] Tomita Y., Ikeda T., Sakata S., Saruwatari K., Sato R., Iyama S., Jodai T., Akaike K., Ishizuka S., Saeki S. (2020). Association of Probiotic *Clostridium butyricum* Therapy with Survival and Response to Immune Checkpoint Blockade in Patients with Lung Cancer. Cancer Immunol. Res..

[166] Duttagupta S., Messaoudene M., Hunter S., Desilets A., Jamal R., Mihalcioiu C., Belkaid W., Marcoux N., Fidelle M., Suissa D. (2026). Fecal microbiota transplantation plus immunotherapy in non-small cell lung cancer and melanoma: The phase 2 FMT-LUMINate trial. Nat. Med..

[167] Fernandes R., Jabbarizadeh B., Rajeh A., Hong M.M.Y., Baines K.J., Ernst S., Winquist E., Ali A.S., Penny S., Figueredo R. (2026). Fecal microbiota transplantation plus immunotherapy in metastatic renal cell carcinoma: The phase 1 PERFORM trial. Nat. Med..

[168] Porcari S., Ciccarese C., Heidrich V., Rondinella D., Quaranta G., Severino A., Arduini D., Buti S., Fornarini G., Primi F. (2026). Fecal microbiota transplantation plus pembrolizumab and axitinib in metastatic renal cell carcinoma: The randomized phase 2 TACITO trial. Nat. Med..

[169] Zhang Y., Shen X., Shen Y., Wang C., Yu C., Han J., Cao S., Qian L., Ma M., Huang S. (2026). Cytosolic acetyl-coenzyme A is a signalling metabolite to control mitophagy. Nature.

[170] Ferrer M., Mourikis N., Davidson E.E., Kleeman S.O., Zaccaria M., Habel J., Rubino R., Gao Q., Flint T.R., Young L. (2023). Ketogenic diet promotes tumor ferroptosis but induces relative corticosterone deficiency that accelerates cachexia. Cell Metab..

[171] Su Z., Liu Y., Xia Z., Rustgi A.K., Gu W. (2024). An unexpected role for the ketogenic diet in triggering tumor metastasis by modulating BACH1-mediated transcription. Sci. Adv..

[172] Hiller J.G., Cole S.W., Crone E.M., Byrne D.J., Shackleford D.M., Pang J.-M.B., Henderson M.A., Nightingale S.S., Ho K.M., Myles P.S. (2020). Preoperative β-Blockade with Propranolol Reduces Biomarkers of Metastasis in Breast Cancer: A Phase II Randomized Trial. Clin. Cancer Res..

[173] Løfling L.L., Støer N.C., Sloan E.K., Chang A., Gandini S., Ursin G., Botteri E. (2022). β-blockers and breast cancer survival by molecular subtypes: A population-based cohort study and meta-analysis. Br. J. Cancer.

[174] De Giorgi V., Grazzini M., Benemei S., Marchionni N., Botteri E., Pennacchioli E., Geppetti P., Gandini S. (2018). Propranolol for Off-label Treatment of Patients with Melanoma. JAMA Oncol..

[175] Li B., Elsten-Brown J., Li M., Zhu E., Li Z., Chen Y., Kang E., Ma F., Chiang J., Li Y.-R. (2025). Serotonin transporter inhibits antitumor immunity through regulating the intratumoral serotonin axis. Cell.

[176] Lee S., Weiss T., Bühler M., Mena J., Lottenbach Z., Wegmann R., Sun M., Bihl M., Augustynek B., Baumann S.P. (2024). High-throughput identification of repurposable neuroactive drugs with potent anti-glioblastoma activity. Nat. Med..

[177] Sanjinez C., Botteri E., Støer N.C., Lukas Löfling L. (2023). Antimuscarinics and lung cancer survival: A Norwegian population-based cohort study. Lung Cancer.

[178] Doz F., van Tilburg C.M., Geoerger B., Højgaard M., Øra I., Boni V., Capra M., Chisholm J., Chung H.C., DuBois S.G. (2022). Efficacy and safety of larotrectinib in TRK fusion-positive primary central nervous system tumors. Neuro-Oncology.

[179] Besse B., Lin J.J., Bazhenova L., Goto K., de Langen A.J., Kim D.-W., Wolf J., Springfeld C., Popat S., Lim D.W.T. (2026). Repotrectinib in NTRK fusion–positive advanced solid tumors: A phase 1/2 trial. Nat. Med..

[180] Wang D.-S., Hu M.-T., Wang Z.-Q., Ren C., Qiu M.-Z., Luo H.-Y., Jin Y., Fong W.P., Wang S.-b., Peng J.-w. (2021). Effect of Aprepitant for the Prevention of Chemotherapy-Induced Nausea and Vomiting in Women. JAMA Netw. Open.

[181] Botteri E., Hjorth S., Conforti F., Bagnardi V., Andreassen B.K., Støer N.C., Bhargava S., Ursin G., Gandini S., Sloan E.K. (2025). Aprepitant use during chemotherapy and association with survival in women with early breast cancer. J. Natl. Cancer Inst..

[182] Olawaiye A.B., Gladieff L., O’Malley D.M., Kim J.-W., Garbaos G., Salutari V., Gilbert L., Mileshkin L., Devaux A., Hopp E. (2025). Relacorilant and nab-paclitaxel in patients with platinum-resistant ovarian cancer (ROSELLA): An open-label, randomised, controlled, phase 3 trial. Lancet.

[183] He X.Y., Gao Y., Ng D., Michalopoulou E., George S., Adrover J.M., Sun L., Albrengues J., Daßler-Plenker J., Han X. (2024). Chronic stress increases metastasis via neutrophil-mediated changes to the microenvironment. Cancer Cell.

[184] Obradović M.M.S., Hamelin B., Manevski N., Couto J.P., Sethi A., Coissieux M.M., Münst S., Okamoto R., Kohler H., Schmidt A. (2019). Glucocorticoids promote breast cancer metastasis. Nature.

[185] Campone M., De Laurentiis M., Jhaveri K., Hu X., Ladoire S., Patsouris A., Zamagni C., Cui J., Cazzaniga M., Cil T. (2025). Vepdegestrant, a PROTAC Estrogen Receptor Degrader, in Advanced Breast Cancer. N. Engl. J. Med..

[186] Conforti F., Pala L., Di Mitri D., Catania C., Cocorocchio E., Laszlo D., Ceresoli G., Locatelli M., Facella F., De Pas T. (2025). Sex hormones, the anticancer immune response, and therapeutic opportunities. Cancer Cell.

[187] Liu S., Wang Z., Su Y., Qi L., Yang W., Fu M., Jing X., Wang Y., Ma Q. (2021). A neuroanatomical basis for electroacupuncture to drive the vagal–adrenal axis. Nature.

[188] Shen G., Ren D., Zhao F., Wang M., Liu Z., Feng X., He Y., Liu X., Ling X., Zhao Y. (2024). Effect of Adding Electroacupuncture to Standard Triple Antiemetic Therapy on Chemotherapy-Induced Nausea and Vomiting: A Randomized Controlled Clinical Trial. J. Clin. Oncol..

[189] Jeon J.Y. (2025). Exercise as a new therapeutic modality in oncology: CHALLENGE trial refines survivorship care. Nat. Rev. Clin. Oncol..

[190] Cheng Y., Xu L., Xia N., Wang J., Yang W., Jin C., Wang R., Qin J., Zhu Y. (2025). Aerobic dance during chemotherapy in patients with breast cancer with cognitive impairment (ADANC): Protocol for an assessor-blinded randomised clinical trial. BMJ Open.

[191] Li J., Li C., Puts M., Wu Y.-c., Lyu M.-m., Yuan B., Zhang J.-p. (2023). Effectiveness of mindfulness-based interventions on anxiety, depression, and fatigue in people with lung cancer: A systematic review and meta-analysis. Int. J. Nurs. Stud..

